# An exploration of patients’ perceptions and coping strategies for LBP

**DOI:** 10.1371/journal.pone.0324859

**Published:** 2025-06-09

**Authors:** Amanda Hall, Andrea Pike, Krystal Bursey, Sameh Mortazhejri, Andrea Patey, Jeremy Grimshaw, Holly Etchegary

**Affiliations:** 1 Primary Healthcare Research Unit, Faculty of Medicine, Memorial University, St. John’s, NL, Canada; 2 Centre for Implementation Research, Ontario Health Research Institute, Ottawa, ON, Canada; 3 Division of Population Health and Applied Health Sciences, Faculty of Medicine, Memorial University, St. Johns, NL, Canada; Aichi Prefectural Mikawa Aoitori Medical and Rehabilitation Center for Developmental Disabilities, JAPAN

## Abstract

**Background:**

Evidence-based guidelines for managing LBP exist but their recommendations are often not used by health professionals in primary care. A key challenge to address this issue is understanding how people understand LBP, how they feel about it, and cope with it – particularly with regard to why they visit their doctors and their treatment expectations. This is important to understand, particularly since physician barriers to following LBP treatment guidelines have centered on patient issues (such as patient demand for imaging).

**Methods:**

This was a qualitative, exploratory study using semi-structured interviews to explore patient perceptions of LBP and their coping strategies, paying particular attention to why patients with LBP in Newfoundland and Labrador (NL) seek care from family physicians and their treatment expectations, especially with regard to imaging. Eligible patients included adults aged 18 + years or older, living in both rural and urban settings in NL, Canada, who had visited their family physician about low back pain within the year prior to the interview. Researchers experienced in applying the Common-sense Model of Self-regulation (CSM), used the model to inform the development of our question guide and as a framework for the data analysis.

**Principal findings:**

We found that new onset, severity, or persistent pain prompted patients to visit their family doctor, primarily to seek advice and/or a diagnosis, or for a referral to imaging or other providers. While patients believed that imaging was essential to understanding the underlying cause of their symptoms or informing their treatment, they were divided about its effectiveness – some felt it was beneficial to their treatment while others reported that it had no effect. We found that patients were unified in their largely negative views regarding prognosis and all experienced a range of negative emotions surrounding their LBP such as fear, stress, frustration, and guilt. We also found wide variation in understanding of cause and use of coping strategies. Patients posited several causes for the pain including injury, overexertion, comorbid conditions, and issues related to posture and sitting, and were split on their thoughts regarding prevention – about half thought it could be prevented, half did not. We found that patients coped with their LBP using a variety of strategies but were often disappointed in the results. Most reported no benefit to visiting their family doctors for their LBP. Some were pleased with their experiences with allied HCPs, noting small, but steady, improvements using recommended exercises but others were generally dissatisfied.

**Conclusion:**

Our exploration of patient views and expectations for low back pain care indicates a mismatch between the care they are looking for and the care they receive. It also suggested a general lack of knowledge about the cause of LBP, the value and usefulness of imaging for its diagnosis and treatment, and poor physician-patient communication.

## Introduction

Low back pain (LBP) is the leading cause of disability globally [1]. While evidence-based guidelines for managing LBP exist [2,3], their recommendations are often not used by health professionals in primary care [4,5] leading to mismanagement of patients’ LBP [6,7]. This includes inappropriate advice regarding rest and activity, liberal prescription of opioid medications, and unnecessary referrals for imaging or surgery [4,5,8,9]. The consequences of this poor care are profound. The direct healthcare costs associated with LBP are a heavy economic burden for health systems and indirect costs (e.g., lost workdays) are even higher [10].

Physician-reported barriers to following LBP treatment guidelines have centered on patient issues. For example, in a systematic review, Hall et al. 2019 [11] found that issues related to perceived patient expectations was one of the most common barriers to following the guidelines for imaging. The most recent guidelines [2,3] all indicate that imaging should be ordered when specific conditions are suspected and/or if the patient is considered a candidate for surgery. In the absence of these conditions, physicians are recommended to educate patients on why imaging is not helpful. Our review found that physicians reported a lack of time to explain diagnosis and prognosis and negotiate patient expectations for imaging as reasons for imaging outside the guidelines. Family doctors also reported that they sometimes ordered images at patients’ request to avoid damage to the doctor-patient relationship, help them gain patient buy-in for treatment advice, and/or reassure patients who struggle with anxiety regarding their LBP. A recent qualitative study with family physicians in Newfoundland & Labrador (NL) noted similar findings [12]. A key challenge to address these issues is to gain a fulsome understanding of why patients visit their doctors for low back pain. What information are they looking for? Are they looking for imaging? If so, why? To answer these questions it is important to understand how people perceive their LBP, how they feel about it, and how they cope with it.

Pain literature suggests that how people think and feel about pain plays a role in their experience of pain, how they cope with it, and even the trajectory of their symptoms [13–16]. Specifically, a wealth of qualitative research from the LPB literature indicates that a person’s beliefs about the cause of their pain affects how they manage their LBP [16–20]. When patients believe their LPB is caused by a damaged or weak spine, for example, they can upregulate protective pain behaviors and avoid valuable activities and movement, thereby acting as a barrier to guideline-concordant care [13,18–22].

This study was completed as part of a larger project developing an intervention to improve overall guideline management of back pain, particularly around recommendations for imaging. While many studies exist about why patients seek care, there isn’t a lot of qualitative literature directly asking patients if, and why, they want imaging as part of their care. This information can be used by researchers to develop tools that help providers better understand and address patient needs.

### Objective

The overall aim of this study is to explore patient perceptions of LBP and their coping strategies, paying particular attention to why patients with LBP in Newfoundland and Labrador (NL) seek care from family physicians and their treatment expectations, especially with regard to imaging.

## Methods

### Design

This was a qualitative, exploratory study using semi-structured interviews to investigate patient perceptions of LBP and care-seeking behavior. The study was approved by the Newfoundland and Labrador Health Research Ethics Authority (File Number 20120484).

### Participants

Eligible participants included adults aged 18 + years or older, living in both rural and urban settings in Newfoundland and Labrador, Canada, who had visited their family physician about acute or chronic low back pain within the year prior to the interview. Those reporting specific causes of low back pain during participant screening were ineligible for participation. Participants were recruited via an advertisement posted on the Primary Healthcare Research Unit’s Twitter and Facebook accounts. The advertisement was also circulated to NL SUPPORT’s patient advisory council who were invited to share the recruitment poster with their contacts. The recruitment period ran from April 7, 2020 to August 17, 2022. We planned to recruit until we reached data saturation (estimating that we would require approximately 15 patients to participate).

### Data collection and analysis

#### Theoretical framework.

We used a social cognition model, the Common-Sense Model of Self-Regulation (CSM) [14], to frame our investigation. Social cognition models are theoretical models demonstrating how psychological processes are thought to influence behaviors. They provide a theoretical background and framework for research by guiding data collection and analysis. This allows researchers to collect and understand data in a more robust and systematic way that doesn’t rely solely on their own inferences. The premise of the CSM is that how patients understand their illness affects how they cope with it. It proposes that when a person experiences a symptom (e.g., LBP), they draw on their existing knowledge, social communications, and personal experience of illness to form two types of illness representations (i.e., beliefs and expectations about an illness). The cognitive representation or “mental picture” includes information related to identity (i.e., the name or label of the illness), cause (i.e., the illnesses’ causal mechanism), timeline (i.e., acute, chronic or cyclical), consequences (i.e., believed consequence of illness), curability/controllability (i.e., whether you can do something about the illness), and prevention (i.e., could/can the illness be prevented). The emotional representation of the health threat refers simply to the person’s emotional responses to the illness and/or treatment. These representations then inform a person’s strategies for coping with the illness which intersect with cognitive and emotional pathways via feedback loops and appraisal of coping strategies as the health threat develops [14]. This model closely aligns with our objective and provides a more comprehensive means to answer our question.

#### Data collection.

Interested patients contacted the research assistant by telephone or email after viewing the study ad. The research assistant briefly explained the study, answered questions, assessed eligibility and emailed eligible patients an information letter. Interested patients then replied to the study contact to schedule an interview. With the permission of the Health Research Ethics Board, written consent was not obtained as participant consent was implied by participation in the interview. Data were collected via semi-structured interviews conducted by two of three female researchers (KB and AP or AH) with Master’s or PhD level training in implementation science or behavioural psychology and experience in qualitative behaviour change and/or LBP research. Interviews were completed over the phone and audio-recorded on secure university-encrypted laptops. Patients were provided a $100 (Canadian Dollar) gift card honorarium (paid for by the research grant supporting this work). All interviews were transcribed verbatim; patients were not provided opportunity to review their transcripts. Brief field notes were taken by one of the interviewers during the interviews as a fail-safe measure in the event of a recording failure but were not used in the analysis.

#### Interview guide.

The interview guide (please see the S1 Appendix: Interview guide) was developed based on the CSM by AH and AP in collaboration with researchers experienced in applying the CSM to qualitative studies (AMP and SM). It was subsequently reviewed by the larger researcher team which included patient partners. It is comprised of questions that covered demographic information and a series of open-ended questions based on the CSM dimensions (e.g., questions about patient understanding of their LBP, how it makes them feel, and what they do to cope with their LBP.

#### Data analysis.

Using the CSM to generate a coding framework, researchers analyzed the data deductively (assigning text to one or more dimensions of the CSM) and inductively (identifying themes within each of the dimensions).

### Deductive analysis

Two female researchers (SM and AMP) experienced in applying the CSM coded the data independently using NVivo 12 software. After coding the first transcript SM and AMP met to discuss and resolve any discrepancies and develop a uniform coding scheme. Further discrepancies were resolved via consensus.

### Inductive analysis

After the data were coded into the CSM dimensions, themes were identified to summarize patterns that emerged from the data coded at each dimension. All themes (with supporting quotes) were reviewed by the second coder (AMP) and members of the larger research team (AP, KB, AH).

## Results

Ten patients (six females, four males) participated in this study; they were between 26 and 50 years of age and either college (n = 2) or university (n = 8) educated. The interviews took between 30 minutes and one hour. Eight of ten patients had no previous interactions with the interviewers; two patients were previous acquaintances of AP or KB). None of the eligible patients refused participation or dropped out. No repeat interviews were conducted. Thematic saturation was assessed after data collection was completed using the method proposed by Guest et al. 2022 [23]. Thematic saturation was achieved at eight interviews after which point no new themes were identified.

We report our findings based on three key areas of inquiry: 1) patients’ understanding of their LBP (cognitive illness representations), 2) how their LBP makes them feel (emotional illness representations), and 3) how they cope with their LBP. Quotes supporting our analysis are included throughout the text of our results section; additional supporting quotes are provided in the S2 Appendix: Additional supporting quotes.

### Cognitive illness representations

#### Identity.

Most patients described their LBP as an ache, stiffness, spasm, tightness, and/or fatigue in their lower back region with or without burning sensations and tingling, pain, or numbness that travelled to the legs. They reported that their LBP could range in severity from discomfort to severe pain and indicated that severity varied from episode to episode. For some, pain was reported to increase over time.

“I have multiple different types of symptoms - it’s a shooting pain…sometimes it will come down to my legs on both sides. Other times it’s like the muscles in my lower back…fatigue and they get really tight.” (P7).

“It aches and there are certain times when, I don’t know what happens, I’m guessing what people call a spasm. Where it, it feels like a knife. It doesn’t happen very often, it’s mostly just an ache. And it’s also coupled with stiffness.” (P3).

“It just got continuously worse and worse.” (P5)

#### Timeline.

Most patients had been experiencing LBP intermittently from two to as many as 9 years. In terms of duration, patients reported that episodes of LBP could last from a few hours to months. Others reported constant pain.

“I started having low back pain about three years ago I’d say.” (P2)

“A couple hours, sometimes a day, sometimes several days, sometimes two or three weeks. It really varies.” (P4).

“I have symptoms from the moment I get up until I go to bed at night.” (P5).

#### Cause.

While most patients were uncertain about the exact cause of their LBP, multiple potential causes were put forward. Some felt their back pain was caused by excessive sitting/standing, poor posture, or simply holding any specific body position for an extended period. Others felt that injury (through sport or accident), overexertion (e.g., heaving lifting), and/or comorbid conditions (e.g., arthritis) played a role in causing their LBP.

“I don’t really know how I got it. It just started happening.” (P5).

“It’s just associated with my condition. I guess from sitting in, in the same position, in the same spot in the same posture for hours at a time.” (P9).

“One day at the gym I just kind of heard a weird noise and once that happened it’s been nonstop, you know.” (P6).

#### Consequence.

Patients reported that LBP limited their activity level by impeding their ability to engage in regular physical activities such as hiking, walking, running, or sports. They also reported difficulties completing daily chores by restricting their ability to move around or bend. Other consequences included difficulties functioning at work requiring sick leave or even job loss, sleep problems, and worsening of comorbidities.

“I used to be a very active person and I’m not anymore because of my lower back pain, you know, especially the shooting pain.” (P7).

“I can’t do normal things. I love to clean my house and I love the feeling I get after, that happens after, the accomplishment, after cleaning is done. And I can’t do it anymore, I can barely wash the dishes. Clean the bathroom? Forget it.” (P3).

“The consequence is definitely work loss. You’re, you’re not enjoying yourself at work. Definitely decrease in your productivity, you know, your willingness to do things. If you’re, if you’re living through pain and your pain is excruciating or increased because of work it’s not gonna make you a very good person to work with or to be productive in the workforce.” (P5).

“Probably affected my sleeping habits the most.” (P1).

“There was points in my life when I thought I was crippled…it was really, really bad. That’s just so depressing and I just laid on my back in my, in my bed and did not move ordering takeout all the time. I’ve gained so much weight…” (P5).

#### Curability/controllability.

Many patients initially believed their LBP would be short-lived but were disappointed when it persisted. Overall, most patients did not believe that their LBP could be cured. However, they were hopeful that their symptoms could be managed and that they would find relief for their pain.

“I thought it would go away or get better, but it started getting worse actually.” (P1).

“I don’t think it can be cured no. I’m at the point where doctors tell me that spinal surgery might have to be an option for me in the future.” (P4).

“It’s controllable I believe it definitely is. I’m at a point now where I’m able to manage it pretty effectively. It doesn’t mean I don’t have bad bouts.” (P6)

#### Prevention.

Patients in our study were split on their feelings about prevention. About half did not believe or were not sure that their pain was preventable. Many were also not optimistic that their symptoms could be prevented from reoccurring. Others believed that LBP could have been prevented in the first place if they had been more vigilant about their movements/behavior. They described several strategies (e.g., taking breaks, going for massage, using a heating pad, trying to be active, being cautious of their posture) to prevent worsening or new episodes of their LBP.

I: Do you think your low back pain could have been prevented in the first place?

R: No, I don’t think so. “(P5)

“I don’t know at this point [if a recurrence can be prevented] because, you know, the damage is already there the damage is done.” (P7).

“You know, my whole life, if I you know as a teenager, you don’t take that stuff seriously, you’re not afraid right. But I mean, if I grew up playing sports and if I did my stretches regularly and if I did sit at the table straight. Or if I used, you know if I didn’t lean back in my car so much you know, I think over time and if I worked at a better pace and stuff like that and ate healthier, then yeah sure. I’m sure that probably could have helped a lot.” (P4).

“I have to move also like I have to get out of the chair pretty consistently if I don’t want to have back pain.” (P10).

### Emotional illness representations

Overwhelmingly, patients reported experiencing a variety of negative emotions surrounding their LBP including, for example, fear, stress, and frustration. Most were afraid their LBP would worsen, potentially leaving them immobile or leading to job loss. Others reported fears that their LBP was caused by a more serious underlying condition. These worries and what they described as constant vigilance of their body movements and symptoms were very taxing for patients leading to frustration and/or irritability. LBP was also reported by some to lead to emotional exhaustion and depression. Finally, patients reported that their LBP and/or the restrictions they felt it placed on their ability to perform work or functional activities caused them to feel helpless or guilty.

“…my biggest fear is having to come off work and go on disability or something like that for a while right.” (P4).

“It’s extremely taxing because my brain is functioning perfectly fine and, you know, it’s just and it’s something that people can’t see so it’s hard to explain to people the severity of it.” (P6).

“It’s pretty stressful. I’m getting older [and] I have to start preparing…or it’s gonna start giving out on me. It’s… stressful having that hanging over your head.” (P10).

“When I gave up my job, I ended up getting really depressed like to the point where I couldn’t get off my couch for like 3-4 days.” (P7).

“It’s pretty infuriating. It’s pretty annoying.” (P2).

“I’m bathing the little guy for example, and he’s in a mood and he’s “No, no!”. And he’s really, really riled up and I get stressed out, then may get angry at him. Normally my patience would be a lot longer, you know what I mean, so it’s that kind of thing as well. It’s not just the physical pain.” (P3)

### Coping strategies

Patients described three main types of strategies for coping with their LBP: information-seeking, self-management, and medical care-seeking.

#### Information-seeking.

Patients commonly reported seeking information about their LBP and how to deal with it. Most often they sought information from allied HCPs (e.g., physiotherapists, chiropractors, massage therapists) to learn about their LBP. They also reported seeking information from their family doctor, internet/social media, and friends and family.

“I: Do you ever seek information about your low back pain and how to deal with it?

P: Yeah, so I went to my GP.” (P2)

“My physiotherapist tells me its because my [low back] muscles are always in protective mode and so the range of movement is not there.” (P3).

“I have an uncle, I have friends, and older friends who have gotten this cortisone needle. The people that I’ve talked to with this with back pain have had a lot of positive results.” (P5).

“Just last night I was on the internet trying to figure out just something that could help. And right now, I’m seeing another massage therapist who thinks that he can help yeah.” (P7).

#### Self-management.

Patients described using a variety of self-management strategies to cope with their symptoms/illness. The most commonly reported strategies ranged from simple actions and/or accommodations enabling them to carry out their day-to-day activities (e.g., adjusting/changing posture, taking breaks, requesting assistance to complete tasks) to those focused on activity (e.g., exercise and stretching), and pain management (e.g., over-the-counter medications, application of hot/cold therapy). Other reported strategies included losing excess body weight and using devices such as a transcutaneous electrical nerve stimulation (TENS) machine or a back brace.

“Well I knew that after if I had stained the patio that I couldn’t tell my son that I was doing a certain thing a day or two days later. So, if he wanted to go somewhere, I would say we have to do this on Wednesday because I’m doing this on Monday. So, it’s almost like we’re organizing your life somewhat because you need to do the things that you, you know, are required to do.” (P8).

“I was doing exercises from the start and I think it definitely helped a lot because like my posture was like the pits before.” (P10)

“I’ll take I usually have Robaxacet at home and if it gets really bad, I’ll take something like that and usually it helps.” (P7)

“I, I try to, you know, always take a hot bath before I go to bed at night to loosen the muscles.” (P6)

#### Medical care-seeking.

All patients reported seeking medical care to cope with their LBP from both family doctors and allied HCPs.

### Family doctors

Just over half of patients reported *regularly* seeking care from their family doctor for LBP. Some patients, however, expressed a reluctance to visit their family doctor right away, sometimes waiting a few days to make an appointment, hoping the symptoms would subside on their own or because they didn’t believe their doctor could help them. Indeed, while seeking care from a family doctor was a common strategy, almost all patients believed that the care they received was not beneficial.

“I usually wait a few days because I’m very lucky my symptoms sometimes ‘go away’ in 2-3 days.” (P9).

“Because I don’t feel like he (family doctor) can do anything further for me. And if I do go to see him, I know that it’s gonna result in a Rx and I don’t feel like that’s the right course of action for me.” (P7).

“I mean not really I mean like there’s not much that he (family doctor) can do except prescribe me some, you know, anti-inflammatories or muscle relaxers or something, you know, and yeah there’s not much more he can do.” (P6).

“In your experience did visiting the doctor have any effect on your low back pain?

I honestly don’t think it did very much for me, no.” (P10).

Factors such as new onset of pain, pain severity and/or persistence prompted patients to seek care from their family doctors. A variety of aims were reported in seeking this care. Most commonly, they were hoping to find out what is causing their pain and/or rule out a serious cause, request imaging, look for advice on how to deal with the LBP, or seek referral to an allied HCP. Requests for surgery, prescriptions refills, pain relief, and generally keeping the doctor apprised of their condition were also reported as reasons for seeking care from a family doctor.

“If it’s something new or if it’s something that’s hanging around for a little bit longer than normal, I go, and I check it out.” (P7).

I would just like to know exactly what it is. Like I’m still not even sure if its like I don’t know, muscle tissue, disc, you know what I mean? Like I’m still been sort of searching for what’s been going on.” (P2)

There was no such thing as ‘yeah we’re gonna go get an x-ray done for you’. There’s nothing like that. I definitely had to push for every x-ray and MRI that I had done. (P5)

“Yeah, I was hoping he’d offer me some kind of guidance as to a chiropractor or something.” (P10)

While only four patients reported seeking care from their family doctor specifically to request imaging, almost all reported receiving referrals for imaging after visiting a family doctor for their LBP. In addition to X-rays, some patients had received MRI or CT scan. Despite this, several patients reported that their doctor did not explain why they were ordering imaging, nor did they explain the results of the imaging.

“I think he just kind like looked at my x-ray, said it was cool and then just sent me off.” (P10).

The patients who had asked for imaging explained that they were hoping that imaging would uncover the root cause of their symptoms. Indeed, most patients believed that all patients with LBP required imaging to help understand the underlying problem causing their pain.

“[Images are required] to make sure you don’t have a disc or fracture or anything with the ligaments.” (P1)

“I wanted to make sure I knew what was wrong and then treat it right away instead of aimlessly trying different stretches and stuff and not actually know what’s on the go.” (P2)

“I guess you’ve got to go and get some imaging. You’ve got to do you’ve got to get something, because I’m pretty much tired of trying to deal with it and doctors saying, you know, there’s nothing we can do.” (P5)

Despite that many of our patients believed imaging to be a necessary part of LBP management, four of the patients who had received imaging reported it to have no effect on their doctor’s approach or treatment plans. Other patients, however, believed their imaging results were used to diagnose the underlying structural problem causing their LBP (e.g., herniated disc) or helped their doctor to prescribe better medications. In addition, one patient felt reassured by having the imaging because it showed no indication of a serious illness. Another patient felt that the results of the imaging helped them emotionally by legitimizing their pain.

“If it wasn’t for the CAT scan that I received, they would have never known about the herniated disc that I had that was causing me severe pain.” (P7)

“I lived with my family…and I was going through this. I think they kind of thought I was just kind of milking it, you know, because my head is fine. I can still laugh, you know, I’m not gonna be, but it was kind of, you know, I remember their faces when I told them the results and they were just shocked, you know. So, but it was I think it helped even emotionally it helped, you know, because you know you’re not going crazy.” (P6).

### Allied HCPs

Most patients had sought LBP care from multiple types of allied HCPs in an effort to manage their LBP. Most commonly, they sought care from physiotherapists, massage therapists, and chiropractors.

“I have been doing a lot of physio.” (P2)

“And right now, I’m seeing another massage therapist who thinks that he can help yeah.” (P7)

“And, you know, I spent thousands and thousands of dollars going to different chiropractors.” (P5)

In terms of treatments, patients explained that their allied HCPs primarily recommended self-management strategies such as stretches or exercises for them to complete at home.

“She [chiropractor] told me every hour at my computer I was like to get up and grab a broom and like put it behind my head and just like stretch my arms out.” (P10).

Patients were divided on their perceptions of the effectiveness of the care they received from allied health professionals. Some were very pleased with their experience or noted small, but steady, improvements while others were generally dissatisfied. Some patients also reported that accessing allied HCPs for their LBP was challenging or impossible because of the cost involved.

“I felt a lot better like almost immediately when she (chiropractor)…she cracked my back, I could just feel the relief that it was pretty nice.” (P10).

“I did physiotherapy, which I don’t do anymore ‘cause that didn’t help me at all.” (P4).

“I don’t understand like why I have to pay to see a massage therapist. I don’t understand that. Why can’t there be someone like a doctor or like any other kind of specialist. You know, if you have heart problems you go see your heart specialist, cardiologist or whatever, you don’t have to pay for it. Why do I have to pay for my physiotherapist? Or why do I have to pay for my massage therapist? I just don’t understand it because it’s a real health problem. It like impacts me a lot more than some other conditions that you can go see a specialist for free so why can’t I get the same kind of care?” (P3).

## Discussion

This study explored patient perceptions of LBP, paying particular attention to *why patients with LBP in NL seek care from family physicians and their treatment expectations, especially with regard to imaging*. We found that new onset, severity, or persistent pain prompted patients to visit their family doctor, primarily to seek advice and/or a diagnosis, or for a referral to imaging or other providers. While patients believed that imaging was essential to understanding the underlying cause of their symptoms or informing their treatment, they were divided about its effectiveness – some felt it was beneficial to their treatment while others reported that it had no effect. Using the CSM allowed a more nuanced exploration of participant perceptions about LBP. We found that patients were unified in their largely negative views regarding prognosis (i.e., they did not believe their LBP could be cured/controlled) and all experienced a range of negative emotions surrounding their LBP such as fear, stress, frustration, and guilt. For example, most were afraid their LBP would worsen or that it was caused by a more serious, underlying condition. However, we also found wide variation in understanding of cause and use of coping strategies. Patients posited several causes for the pain including injury, overexertion, comorbid conditions, and issues related to posture and sitting, and were split on their thoughts regarding prevention – about half thought it could be prevented, half did not. In terms of coping, we found that patients coped with their LBP using a variety of strategies but were disappointed in the benefits realized from these strategies. For example, most reported no benefit to visiting their family doctors for their LBP. Some were pleased with their experiences with allied HCPs, noting small, but steady, improvements using recommended exercises but others were generally dissatisfied.

### Comparison with the literature

Our findings regarding why patients seek care from their family doctors for LBP are consistent with previous research. For example, Lim and colleagues [24], in a systematic review of 41 studies conducted in 13 countries, found that patients want more information about LBP from their providers, including its cause and pathology as well as a desire for a diagnosis and information on prognosis.

Lim’s review also found that patients desire imaging [24]. In contrast, we found that less than half of our participants had visited their doctor specifically to request imaging. That being said, almost all reported receiving an imaging referral and our results indicate a general lack of awareness about the low value of imaging in the diagnosis and treatment of non-specific LBP. This is consistent with other studies that have assessed patient and public beliefs about imaging [25,26]. For example, patients we interviewed believe imaging was helpful to diagnose their condition, inform their treatment, and legitimize their pain. This is consistent with previous research showing that patients believed imaging would confirm their physician’s diagnosis, direct their treatment plan [24,27] and provide evidence that their pain is real [28].

The patients we interviewed reported variable causes for their LBP. Likewise, a recent scoping review [29] of 81 quantitative studies completed in 39 countries explored causal beliefs about LBP and identified 308 unique causal beliefs grouped into 15 distinct categories. The most prevalent type of causal beliefs found amongst 56% of the studies they reviewed were those related to the belief that LBP is caused by some form of structural injury or impairment to the anatomy of the back (e.g., muscle strains or disc problems) that can be revealed via imaging. Beliefs that LBP is caused by lifting and/or bending (32%) and mental stress or other psychological factors (30%) were also among the top three types of beliefs noted in the literature. Patients in our study also reported causes related to both injury/impairment and lifting/bending. We did not, however, find causes related to stress or other psychological factors.

Interestingly, most patients in the current study reported little to no benefit from visiting their family doctors. Some felt their family doctors were dismissive and others reported that their physicians didn’t explain why they were ordering images or the results of the imaging. This suggests poor communication between physicians and patients making it difficult for physicians to provide information that meets the needs of their patients. In a systematic review of 20 quantitative and qualitative studies of patient expectations for back pain care conducted in 7 countries, Verbeek noted a similar gap in patient expectations and their experience with healthcare providers [30]. More recently, a study comparing physician assumptions and patient preferences regarding back pain showed that patients most desire an explanation of the cause of their back pain but that physicians underestimate this importance and overvalue the importance of diagnostic imaging to patients [31]. In the end, patients are looking for providers who listen to them and communicate well – particularly when it comes to explaining their condition and/or pain and providing instruction or advice [30].

### Implications for research and practice

Current evidence-based practice for LBP advises against imaging unless red flag conditions are present (such as history of cancer, unexplained weight loss, fever, recent infection, signs of nerve damage, abnormal reflexes, etc.) [2,3]. Imaging without the presence of red flags is considered to be low value use and may encourage persistent unhelpful patient beliefs. On the contrary, avoiding low-value imaging may set patients up for more appropriate expectations. For example, Kiel 2020 showed that among patients who had experienced back pain in the last 12 months, a lack of prior imaging was associated with a decreased expectation of imaging [32]. Choosing Wisely Canada has adapted information from the US Choosing Wisely campaign to reduce low-value care for LBP to provide physicians and patients guidance on when to use imaging and why, as well as advice on how to best manage LBP [33,34].

Our results indicated that while only some patients requested imaging for their LBP, almost all had been referred for imaging by their family doctors. Patients also did not feel their family physician explained why they ordered an image or the results of the image. Additionally, we noted that some patients felt the image would legitimize their pain. We believe these results point to a need for improved patient-provider communication. Recent research by Sharma and colleagues [35] demonstrated that when provided with the proper information, the public can be educated on how these diagnostic tests are not beneficial and can even be harmful for those with uncomplicated LBP. Physicians need more effective ways to educate patients about LBP and the role of imaging and alternative ways to validate patients’ pain without the use of images. Providing space within the clinical encounter to acknowledge and validate the emotional difficulties associated with LBP could be an effective response to patients’ needs for validation and emotional support.

While we did not interview physicians in this study, the literature suggests that physicians believe patients want imaging [11,31]. This indicates a mismatch between the needs the patients described in this study (i.e., diagnosis, prognosis, management etc.) and physicians’ beliefs about what patients want from their visit. This mismatch is an important finding that may help guide the design of future interventions to reduce imaging for LBP. Improved communication between doctors and patients would elicit more accurate patient expectations, potentially reduce low-value imaging for uncomplicated LBP, and hopefully, better address patients’ needs. Also, it is important for us to understand the prevalence of ordering due to patient requests. Identifying the magnitude of this problem can help researchers understand how to better develop targeted interventions to improve the uptake of imaging guidelines for LBP.

Our study did not intend to assess differences in patient’s understanding of their back pain and treatment-seeking based on the duration of their back pain (acute vs chronic). Rather, we see these results as beneficial for the development of patient education materials for all patients experiencing back pain. However, assessing differences in acute and chronic populations may be an area for future research to determine if tailoring education to these groups would be beneficial.

### Limitations

This is the first study to assess why patients in NL visit their GPs for LBP, their expectations for that care, and their expectations and perspectives related to imaging. While our study provides important insights, it is limited in several ways. First, our sample was restricted to English speaking individuals. While NL is predominately English speaking, limiting our sample in this way may have excluded more diverse perspectives. In addition, all interviews were conducted with patients who had already sought care for LBP from their family doctors. Therefore, their interviews relied on their memory and past experiences and it is possible their recollections were affected by recall bias. We attempted to mitigate this issue by interviewing patients who had visited their family doctors about their back pain within the preceding 12 months. The sample was also missing the perspectives of those who do not visit healthcare providers as part of LBP management. It is acknowledged their beliefs and perceptions may be different from patients who do. Finally, we did not assess potential regional differences in patient attitudes or access to care or any cultural differences that may influence patient perceptions of their LBP and/or their expectations for treatment. We acknowledge that, if present, such differences could provide a more nuanced understanding of patient expectations that could inform regionally tailored interventions in LBP management.

## Conclusion

Our exploration of patient views and expectations for low back pain care indicates a mismatch between the care they are looking for and the care they receive. It also suggested a general lack of knowledge about the cause of LBP, the value and usefulness of imaging for its diagnosis and treatment, and poor physician-patient communication. Previous research has described patient information needs and indicated that patients can be successfully educated about the appropriate use of imaging. Moving forward, interventions focused on developing communication between patients and physicians should be paramount. Patients want to know they are being heard and their experience and pain is being validated. Physicians need better tools to help them educate patients on appropriate management of LBP and appropriate use of imaging.

## Supporting information

S1 Appendix Interview guide. (DOCX)

S2 Appendix Additional supporting quotes. (DOCX)
