# Supplementary material for: An exploration of patients’ perceptions and coping strategies for LBP
Source: PLoS One. 2025 Jun 9;20(6):e0324859. doi: 10.1371/journal.pone.0324859 (PMC12148076; doi:10.1371/journal.pone.0324859)
Supplement: S1 Appendix — (DOCX) [file pone.0324859.s001.docx]

**S1 Appendix: Interview guide**

Thank you for participating in this interview. Information you provide in this interview will be identified by a study ID code and will not be associated with your name or other personal identifying information. The purpose of the interview is to understand what you know about low back pain (LBP), and how you manage its symptoms. We recently conducted a survey of the Newfoundland & Labrador public to understand people’s low back pain experiences. We are now hoping to better understand what people do when they experience low back pain, by seeking more detail about and validation of survey responses. The information you provide here, including your thoughts and comments will not be shared with your family doctor, or other healthcare providers. There’s no right/wrong way to answer the questions; however, we are looking for honest answers. It helps us to understand the current concerns of patients.

I would like to start with some general questions:

1. Gender?
2. Your age?
3. Your highest level of education?

Now I would like to talk to you about your experience with having low back pain and how you manage that. For the purpose of this interview, we are going to focus on uncomplicated LBP, which means LBP that isn’t caused by a specific condition such a fracture or an infection.

1. Could you please tell me about your symptoms when you had LBP? What were they like? (Prompts: The duration of your symptoms? The severity of your symptoms? How did it make you feel?)
2. What do you think caused your LBP? (Prompts: biological causes, emotional causes, environmental causes)
3. What are/were the consequences of LBP on you? (Prompts: did it make you less effective at work, did you need to take time off from work, did it result in long-lasting complications?) How does it make you feel? Does it worry you? What are your most important concerns about it?
4. Do you think your LBP can be cured or controlled? How?
5. Do you believe it could be prevented in the first place? Is there anything you could do to prevent it from happening again?
6. Do you ever seek information about LBP and how to deal with it? If so, where do you look for information? (e.g. internet, family, friends, doctor, physiotherapist, …)
7. What is your usual approach to managing your LBP? Does it work?
8. Do you usually visit a doctor for LBP?

a) If yes,

1. When do you go? (Prompts: As soon as the symptoms appear (any symptoms)? Or it takes you a few days before going to doctor?)
2. What are you aiming for when you see a doctor? (Prompts: For a prescription? For imaging? For reassurance? For a referral to a specialist?)
3. What factors would make you go see a doctor? (Prompts: For example, the severity of the symptoms? The duration of the symptoms? The friends/colleagues’ advices? The fear of complications?)
4. In your experience, did visiting the doctor have any effect on your LBP?

b) If no,

1. Why? (Prompts: because of factors related to your time, your doctor, your LBP)
2. In your experience, has your LBP (the duration, the severity) been affected by not seeking a doctor?
3. It seems that some patients with LBP visit their doctors to ask for imaging. We would like to know your thoughts on this. Do you think all patients with LBP require imaging as well? Could you please explain your reasons (if yes, why, if no, why?)
4. If you visited a doctor for your last LBP, did you ask for imaging to be done?
5. Did the doctor explain to you why you don’t need an imaging?
6. Did you share your concerns with your doctor? (e.g. that you think if you don’t get imaging, it can’t be diagnosed/treated properly)
7. In case you did an imaging, did it have any effect on your doctor’s approach/treatment plan?

Thank you. Is there anything else about the management of your low back pain you would like to talk to me about that I haven’t covered in this interview?
