# Supplementary material for: An exploration of patients’ perceptions and coping strategies for LBP
Source: PLoS One. 2025 Jun 9;20(6):e0324859. doi: 10.1371/journal.pone.0324859 (PMC12148076; doi:10.1371/journal.pone.0324859)
Supplement: S2 Appendix — (DOCX) [file pone.0324859.s002.docx]

**S2 Appendix: Additional supporting quotations to support thematic analysis of the data**

**Table 1: Thematic analysis of patient’s cognitive illness representations with supporting quotations**

| **Cognitive illness representations** | **Theme (N)** | **Supporting quotations** |
| --- | --- | --- |
| **Identity** | LBP is an ache, pain, stiffness, spasm, tightness, and/or fatigue in the lower back region with or without burning sensations and tingling, pain, or numbness that travels to the legs. (N=10) | “I have multiple different types of symptoms - it’s a shooting pain…sometimes it will come down to my legs on both sides. Other times it’s like the muscles in my lower back…fatigue and they get really tight.” (P7).  “It aches and there are certain times when, I don’t know what happens, I’m guessing what people call a spasm. Where it, it feels like a knife. It doesn’t happen very often, it’s mostly just an ache. And it’s also coupled with stiffness.” (P3).  “it’s like almost like a burning sensation in my lower back” (P10)  “I get severe tingling in my leg and I lose sensation in my toes… My whole back actually spasms, but more to the point my lower back gets very inflamed. I’m unable to walk with it. I have very limited mobility in general.” (P6) |
|  | Severity of pain varied from discomfort to severe pain from episode to episode for most patients. (N=10) | “There are certain times when, I don’t know what happens, I’m guessing what people call a spasm. Where it, it feels like a knife. It doesn’t happen very often, it’s mostly just an ache.” (P3)  “The pain…can range from…two out of 10 to sometimes 8 or 9 out of 10.” (P4)  “It does vary…like today I woke up well my back pain woke me up from bed. I’m able to (through the physio and different things I’ve learned) I’m able to loosen it a little bit so that I’m mobile. That’s pretty standard that happens pretty much every day and then when it’s really bad it’s two weeks. I can’t move I can’t do anything.” (P6) |
|  | For some patients the pain has increased over time. (N=2) | “It just got continuously worse and worse.” (P5).  “It used to just be, like it would never affect anything that I was doing, like when it started off. But over the last I’d say like a year and a half, like bending and everything, like its been a constant pain.” (P2) |
| **Timeline** | Participants had been experiencing LBP intermittently from two to as many as 9 years. (N= 8) | “I: How long have you had these symptoms?  P: I want to say 2-2 ½ years at least.” (P10)  “Well, I started having low back pain about three years ago I’d say.” (P2)  “I've been having symptoms since 2010, 2011” (P4)  “I have had low back pain for approximately four years.” (P3) |
|  | Episodes of LBP could last from a few hours to months. For some the pain was constant.  (N=8) | “A couple hours, sometimes a day, sometimes several days, sometimes two or three weeks. It really varies.” (P4)  “Well, it varies. It could be up to a day or it could be a long 6-8 hours depending on what I’ve done to cause the pain.” (P8)  “Usually, it probably lasts like 1-2 days.” (P9)  “But over the last two, I’d say like a year and a half, like bending and everything, like its been a constant pain.” (P2)  “I have symptoms from the moment I get up until I go to bed at night right now in my life.” (P5) |
| **Cause** | Most patients were uncertain about the exact cause of their LBP, but all suggested multiple potential causes related to sitting and posture, injury, or comorbid conditions. (N=10) | “I have a great aunt who died with rheumatoid arthritis now I don’t have rheumatoid arthritis, but it’s there’s I may have a, a greater risk…but… I don’t really know how I got it. It just started happening.” (P5).  “I must have done something strange.” (P3)  “It’s just associated with my condition. I guess from sitting in, in the same position, in the same spot in the same posture for hours at a time.” (P9).  “One day at the gym I just kind of heard a weird noise and once that happened it’s been nonstop, you know.” (P6).  “Usually, it comes with like really long extended periods of poor posture.” (P10) |
| **Consequence** | Patients reported that LBP limited their activity level by impeding their ability to engage in regular physical activities. (N=7) | “I used to be a very active person and I’m not anymore because of my lower back pain, you know, especially the shooting pain.” (P7)  “I run almost everyday of my life and I haven’t run in a really long time. Like over a year, its been over a year since I have tried to run. Cause it will hurt, like I can run, I can do it for five or six minutes but then a couple hours later I can’t move. I literally cannot move my body.” (P3)  “I can’t walk even though it’s good for the to relieve any or to loosen the muscles. I just can’t do it.” (P6) |
|  | Patients reported that LBP made it difficult for them to complete daily activities and chores by restricting their ability to move around or bend. (N=8) | “I can’t do normal things. I love to clean my house and I love the feeling I get after, that happens after, the accomplishment, after cleaning is done. And I can’t do it anymore, I can barely wash the dishes. Clean the bathroom? Forget it.” (P3).  “I find that bending over is difficult. Putting on socks some days is difficult, lying down is fairly uncomfortable...sitting in a chair” (P4)  “Okay I’ll just be blunt I couldn’t go to the bathroom correctly (laugh) you know, it, it was really, really bad.” (P5)  “So, it restricts my I would say family life for a day or two and it restricts what I, what I choose to do and what I believe I can’t do.” P8 |
|  | Patients reported difficulties functioning at work leading to sick leaves or even job loss. (N= 5) | “It makes my job really hard. There's been a couple times at work where I've had a muscle spasm or something, and I mean one time they had to get a medic and had to give me a valium and stuff at work right.” (P4)  “I don’t have a job anymore I can’t, I can’t work in the job that I had. I had to quit and then I left and went to another job thinking that would be better and then I had to give up that job as well.” (P7)  “The consequence is definitely work loss. You’re, you’re not enjoying yourself at work. Definitely decrease in your productivity, you know, your willingness to do things. If you’re, if you’re living through pain and your pain is excruciating or increased because of work it’s not gonna make you a very good person to work with or to be productive in the workforce.” (P5). |
|  | LBP led to sleep problems (N=1) | “Probably affected my sleeping habits the most.” (P1) |
|  | LPB or its treatments led to other health problems and major procedures (N= 3) | “There was points in my life when I thought I was crippled…it was really, really bad. And, and that’s just so depressing and I just laid on my back in my, in my bed and did not move ordering takeout all the time. I ballooned up I’ve gained so much weight… for a while my sugars were really high [affecting my] Type 2 diabetes.” (P5) |
| **Curability/controllability** | Some patients initially believed their LBP would be short-lived but were disappointed when it persisted. (N=2) | “I thought it would go away or get better, but it started getting worse actually.” (P1).  “Because [at first] I was more like maybe this will heal on its own kind of thing.” (P2) |
|  | Most patients did not believe or were unsure if their LBP could be cured but were hopeful that their symptoms could be managed and that they would find relief for their pain. (N=6) | “I don't think it can be cured no. I'm at the point where doctors tell me that spinal surgery might have to be an option for me in the future.” (P4).  “This is chronic. I think I’m gonna end up with this for the rest of my life.” (P6).  “I don’t know…. if it could be cured or not. I don’t know if it requires surgery like I haven’t really I haven’t researched that enough to know.” (P10)  “I am hoping…I have an uncle, I have friends and older friends who have gotten this cortisone needle. I’m not sure what’s in it but it’s, you know, an epidural needle that you get in your spine.” (P5)  “I’d like to think that eventually I would be able to, to feel 100%.” (P2). |
|  | Some patients believe their LBP can be cured or controlled (N=4) | “[It could be] controlled if I had the money to be seeing a chiropractor every week (laugh) and getting adjustments.” (P10).  I: So you think your low back pain could be cured or controlled?  P: Yes. (P3) |
| **Prevention** | Some patients did not believe or were unsure if their LBP could have been prevented. (N=5) | “I: Do you think your low back pain could have been prevented in the first place?  R: No, I don’t think so. " (P5)  “I: Do you think your low back pain could have been prevented in the first place?  R: I don’t know, maybe? I don’t know.” (P3)  “I think there’s an underlying problem there which is probably what I originally had, but with the severity in the last three years I think that could have been prevented.” (P6) |
|  | Some patients also believed that there was no way to prevent their symptoms from happening again. (N=3) | “At this point, no. Prevent, no. Fix, yes! But yeah, prevent no.” (P3)  “So, I think I can manage it most of the time and control it, but I can’t prevent it entirely, sometimes the littlest thing can trigger it right.” (P4)  “I: Do you think there’s anything you could do from prevent it from happening again?  R: I don’t know at this point because, you know, the damage is already there the damage is done.” (P7) |
|  | Some patients believed that LBP could have been prevented in the first place if they were more vigilant about the possible causes. (N=5) | “You know, my whole life, if I you know as a teenager, you don't take that stuff seriously, you're not afraid right. But I mean, if I grew up playing sports and if I did my stretches regularly and if I did sit at the table straight. Or if I used, you know if I didn't lean back in my car so much you know, I think over time and if I worked at a better pace and stuff like that and ate healthier, then yeah sure. I’m sure that probably could have helped a lot.” (P4)  “I: Do you think or do you believe that it your back pain could have been prevented in the first place?  R: Absolutely, absolutely.” (P7)  “I’m not really sure when it first got developed but I have a few ideas of maybe when. So maybe if I was a bit smarter in what I was doing, perhaps?” (P2) |
|  | Patients described several strategies (e.g., taking breaks, going for massage, using heat pad, trying to be active, being cautious of the posture) to prevent worsening of symptoms or occurrence of new episodes of illness. (N=5) | “I’m just pretty cautious of just what position my back is in, not playing any sports right now.” (P2)  “I have my heating pad that’s, that’s pretty much my prevention and trying to be somewhat active.” (P6)  “I have to move also like I have to get out of the chair pretty consistently if I don’t want to have back pain.” (P10) |

**Table 2. Thematic analysis of patients’ emotional illness representations with supporting quotations.**

| **Theme (N)** | **Supporting quotations** |
| --- | --- |
| Patients reported experiencing a variety of negative emotions and stress surrounding their low back pain and were afraid their LBP would worsen, potentially leaving them immobile or leading to job loss, or that their LBP was caused by a more serious underlying condition. (N=10) | “It’s pretty stressful. I’m getting older, right, like I have to start preparing…or it’s gonna start giving out on me. So, like it’s kind of stressful always having that hanging over your head, I guess.” (P10)  “It adds to the stress that you everyday would normally feel. It just piles up on top of it.” (P3)  “You know I don’t want to be in a position in 5-10 years’ time where I’m unable to be mobile. And, you know, that’s, that’s very frightening.” (P6)  “I’m married, got a home in …, I got a little baby on the way, we had an ultrasound this morning. So, you know I’m a family guy now and I’m only 37 so my biggest fear is having to come off work and go on disability or something like that for a while right.” (P4)  “So emotionally, you know, what I worry about is like am I ever gonna get another job and what, what can I do? What limitations am I gonna [have]? You know, like what’s, what’s my future in life, you know, so many different worries? I could go on and on.” (P5)  “I was scared that it had to do with kidneys, so I think I was terrified that, cause I did have some kidney problems when I was young and I was scared they were back.” (P1) |
| Patients’ symptoms, worries, and constant vigilance about their body movements were very taxing for patients leading to emotional exhaustion, depression, frustration and/or irritability. (N=8) | “I just wanna give up sometimes. Like I don't wanna do anything.” (P4)  “I want to say that it’s like unmotivated to do anything like and it’s like I want to sit down for the day and do nothing.” (P10)  “I couldn’t just mentally take it anymore like that’s towards the end of before I moved home. I just couldn’t go to work because of back pain. Emotionally I couldn’t handle it, you know, like it was just it was too much to get me out of bed to actually do it. I didn’t want to feel it anymore, you know, to that point that’s not a good point.” (P5)  “Like when I gave up my job, I ended up getting really depressed like to the point where I couldn’t get off my couch for like 3-4 days.” (P7)  “It’s extremely taxing because my brain is functioning perfectly fine and, you know, it’s just and it’s something that people can’t see so it’s hard to explain to people the severity of it.” (P6)  “I’m bathing the little guy for example, and he’s in a mood and he’s “No, no!”. And he’s really, really riled up and I get stressed out, then may get angry at him. Like normally that wouldn’t happen. Normally my patience would be a lot longer, you know what I mean, so it’s that kind of thing as well. It’s not just the physical pain.” (P3) |
| Patients reported that their LBP and/or the restrictions they felt it placed on their ability to perform work or functional activities caused them to feel helpless or guilty. (N=4) | “I guess I feel guilty over it myself personally when it comes to work and, and going to the doctor because it’s not really overtly provable.” (P6)  “I can’t even sugarcoat it, it’s terrible. Like it’s, you know, I’m someone who worked my entire life and now I can’t and that that’s really hard… Like some days like you feel like I feel completely helpless, you know, but I mean I try not to let that that feeling stick around for too long. But a lot of times it creeps back in and you feel like, you know, well there’s no help and I mean there’s nothing I can do and, you know, it’s a pretty helpless feeling.” (P7)  “[My symptoms] first of all make me feel restricted like I, you know, as soon as I try to get up I know, you know, I overdid it that day or the day before. It makes me feel old.” (P8) |

**Table 3: Thematic analysis of patient-reported coping strategies with supporting quotations**

| **Coping strategy** | **Theme (N)** | **Supporting quotations** |
| --- | --- | --- |
| **Information-seeking** | Most participants had actively sought information about their back pain (N=9) | “I: Do you ever seek information about LBP and how to deal with it?  P: Yeah, so I went to my GP” (P2)  “I: Do you ever seek information about your low back pain and how to deal with it?  P: Yeah, all the time.” (P4)  “I: Do you ever seek information about low back pain and how to deal with it? For example, you mentioned you were talking about your uncle, what about your friends or the Internet, physiotherapists, doctors?  R: Oh, I’ve did it all.” (P5)  “I: Do you ever seek information about your low back pain and how to deal with it like you could like this could be family, friends, Internet, physiotherapists, doctors?  R: Yes, I’ve done a lot of those things.” (P7) |
|  | Allied healthcare providers (physiotherapists, chiropractors, massage therapists) were the most common sources of information for almost all patients. Doctors, internet and friends and family were the other popular sources. (N=9) | “My physiotherapist tells me its because my [low back] muscles are always in protective mode and so the range of movement is not there.” (P3).  “My doctor told me, and the chiropractor told me it’s very common with students just because you’re constantly hunched over a desk for like 5-8 hours/day.” (P10).  “My own google searches and stuff, and looking stuff up, probably not recommended but you know. I’ve looked into it all, yeah.” (P2).  “I asked my friend and they recommended to do some massages.” (P1).  “I’m in some Facebook groups about back pain and read comments and stuff.” (P4).  “I have an uncle, I have friends, and older friends who have gotten this cortisone needle. The people that I’ve talked to with this with back pain have had a lot of positive results.” (P5).  “Just last night I was on the internet trying to figure out just something that could help. And right now, I’m seeing another massage therapist who thinks that he can help yeah.” (P7). |
| **Self-management** | Actions and/or accommodations enabling patients to carry out day-to-day activities by adjusting/changing posture, taking breaks, requesting assistance to complete tasks, etc. (N=7) | “Sometimes you just got to fight through it too right. And do what you can to get through it, do it a certain way, if you got to lift something get your buddy to help ya, and just do whatever you can to get through it right.” (P4).  “Living with it like figuring out my new life, you know, being able to cope with it. You know trying to figure out what I can do as a career going forward to be able to that I can work at with my low back pain yeah.” (P7).  “Well, I knew that after if I had stained the patio that I couldn’t tell my son that I was doing a certain thing a day or two days later. So, if he wanted to go somewhere, I would say we have to do this on Wednesday because I’m doing this on Monday. So, it’s almost like we’re organizing your life somewhat because you need to do the things that you, you know, are required to do.” (P8).  “I try to catch myself on slouching.” (P10) |
|  | Exercises/stretches (N=8) | “I used to work in an office job so I used to be able to just stand up and stretch, do what I can to mitigate the pain while sitting down in a chair all day.” (P2)  “Stretching, and strength exercises such as squats. Body weight exercises, some ab, abdominal exercises but the main share of what I do is stretching. Walking, and I don’t mean like slow grocery store walking I mean just walking at a nice pace. A good pace that gets your heart rate going.” (P3)  “I've been doing my exercises suggested to me by my health professionals and working on my core and stuff like that to try to get in shape right.” (P4)  “I try to stretch do the stretches that the physio therapist had shown me. I try to do those multiple times/day.” (P6)  “I was doing exercises from the start and I think it definitely helped a lot because like my posture was like the pits before.” (P10) |
|  | Taking over the counter medications (n=6) | “I drink water and I take pain killers and muscle relaxers.” (P4)  “I’ll take I usually have Robaxacet at home and if it gets really bad, I’ll take something like that and usually it helps.” (P7)  “I was for a while taking like glucosamine and things like that for bones and joints and that was kind of nice.” (P10) |
|  | Hot/cold therapy (N=5) | “I try to use Bio freeze in between like nice cold showers or hot showers whatever I, I think is, is, you know, needed at that time.” (P8)  “If it’s really bad I’ll use hot or cold depending on how it feels.” (P7)  “I, I try to, you know, always take a hot bath before I go to bed at night to loosen the muscles.” (P6) |
|  | Weight loss (N=1) | “I went on a keto diet and tried to lose weight. And I did lose up to 80 lbs.” (P5) |
|  | Devices/braces (N=2) | “I have my own TENS machine.” (P6)  “I wear a back brace when I have to, I try not to let my back get dependent on that. But some days when I know I really need it I will wear it or like when I know I'm doing really hard work for a couple hours, I will put it on to try and keep my back straight.” (P4) |
|  | Resting (N=2) | “Like sometimes it’s just when I get home it’s like I just lie down for a few minutes.” (P10)  “I can just mitigate really strong pain by just lying down, you know.” (P5) |
| **Medical care seeking – Family doctor** | Just over half of patients reported regularly seeking care from their family doctor for LBP. (N=6) | "I: Do you usually visit your GP now for your low back pain would you say or?  R: All the time, all the time yeah." (P6)  “I: Would you say you usually visit a doctor for your low back pain? When you’re having new episodes etc.?  P: Yeah, yeah. Usually to follow-up and to renew prescriptions and stuff right… At first it was like you know doctor what’s wrong with me, you know I’m broken. And then it was like okay, try this and this, and then I would go back and it would be…’okay, we’re gonna have to do x-ray’. Okay do the x-ray, ‘okay we need more information, we’re gonna have to do MRI’. So, at first it was the development of figuring out what’s on the go, for so many appointments right, and then following that it’s more or less now how have you been, here’s a slip for a new bottle of pills type of thing.” (P4)  “Yeah, so I have gone to my GP probably about three or four times regarding my LBP.” (P2) |
|  | Some patients waited for a few days before visiting their doctors hoping that the symptoms would heal on their own or because they believed their doctors would not be able help them. (N=8) | “It definitely took me a while to go because I was more like maybe this will heal on its own kind of thing.” (P2).  “I usually wait a few days because I’m very lucky my symptoms ‘go away’ in 2-3 days.” (P9).  “It would just be a waste of time for me, ‘cause I know what the doctor is gonna do and the doctor knows.” (P4).  “I don’t think anything right now would, would because, you know, go to my doctor right now because they can’t really do anything right now.” (P5).  “Because I don’t feel like he (family doctor) can do anything further for me. And if I do go to see him, I know that it’s gonna result in a Rx and I don’t feel like that’s the right course of action for me.” (P7).  “[Because] he (family doctor) will tell me probably what he told me when my child was born, you know, ice it or put some heat on it. Take you time, you know, bend your knees when you’re lifting things of that nature.” (P8). |
|  | Almost all patients believed that they had not received much benefit from visiting their family doctors. (n=7) | “He (family doctor) didn’t do anything. He was very dismissive. He tried some drugs with me, and I didn’t like them and the way that they affected me. They didn’t decrease my back pain well like I said I, I didn’t take them for very long either.” (P5).  “I mean not really I mean like there’s not much that he (family doctor) can do except prescribe me some, you know, anti-inflammatories or muscle relaxers or something, you know, and yeah there’s not much more he can do.” (P6).  “In your experience did visiting the doctor have any effect on your low back pain?  I honestly don’t think it did very much for me, no.” (P10).  “I ended up having to switch GPs because of the lack of care that I was getting with the other one.” (P7). |
|  | New onset of pain, pain severity and/or persistence prompted patients to seek care from their family doctors. (N=6) | “If it’s something new or if it’s something that’s hanging around for a little bit longer than normal, I go, and I check it out.” (P7).  “If its severe enough, I will try to get down there right away because I realize that I can’t really do much in the condition that I’m in currently. And duration as well. Like when it didn’t go away after a while.” (P2).  “If I’ve, if I’ve been dealing with severe pain for a week let’s say depending on how it is I will then go to see him.” (P6). |
|  | Some patients sought care from their family doctor to find out what is causing their pain and/or rule out a serious cause. (N=4) | “I would just like to know exactly what it is. Like I’m still not even sure if its like I don’t know, muscle tissue, disc, you know what I mean? Like I’m still been sort of searching for what’s been going on.” (P2)  “I want him to check just to make sure, make sure that there’s no severe swelling or anything, you know, those types of things.” (P6) |
|  | Some patients visited their family doctor to seek a referral for imaging. (N=4) | “I wanted to do the x-rays …to make sure there was nothing wrong with my kidneys.” (P1)  “I’ve actually lobbied for an MRI now. Just because I really just don’t know what’s on the go. “ (P2)  “There was no such thing as ‘yeah we’re gonna go get an x-ray done for you’. There’s nothing like that. I definitely had to push for every x-ray and MRI that I had done.” (P5) |
|  | Some patients sought care from their family doctor for advice on how to deal with their LBP. (N=3) | “It’s, it’s anything that he can possibly do to help me whether it is reassurance or if there’s something new that I don’t know about or an exercise he suggests.” (P6)  “Yeah, I was hoping he’d offer me some kind of guidance.” (P10) |
|  | Some visited their family doctor to seek referral to allied HCPs. (N=2) | “Because I knew that if I did need to see a physiotherapist or massage therapist or maybe someone else, chiropractor, I don’t know, then I would probably need a referral.” (P3) |
|  | Some patients reported that their doctor did not explain why they were ordering imaging, nor did they explain the results of the imaging. (N=3) | “No doctors, GPs would never give you anything like that.” (P5). |
|  | Patients believe images are required to understand the underlying problem causing their LBP. (N=7) | “To make sure you don’t have a disc or fracture or anything with the ligaments.” (P1)  “‘Cause I just like so worried about what is actually wrong with my back. Like I wanted to make sure I knew what was wrong and then treat it right away instead of aimlessly trying different stretches and stuff and not actually know what’s on the go.” (P2)  “And I don’t know if what I’m dealing with now is all part of the same issue, you know, like I don’t know if I have other slipped discs since (laugh), you know, and these are different. Or are they the same I just don’t know? So, it’s nice to know the area nice to know that it’s consistent or new or, you know, something else is going on. Yeah, I think it would be important.” (P6) |
|  | Imaging may help to prove to others that the LBP is legitimate. (N=1) | “I often wonder if because it’s such a hidden thing and everybody, you know, says that they have back pain I, I often wonder if it’s kind of just proving that, I’m being honest.” (P6) |
|  | Some patients who had received imaging reported it to have no effect on their doctor’s approach or treatment plans but others felt images were used to diagnose and treat their LBP or were otherwise helpful to them (e.g., for reassurance). (N=8). | “If it wasn’t for the CAT scan that I received they would have never known about the herniated disc that I had that was causing me severe pain.” (P7)  “I had it done just to have it out of the way and like make sure there was no like anything actually terribly wrong, but I’m glad I didn’t have like a, like a pinched nerve or a slipped disc or something that was a relief. So yeah, I’m definitely happy I had one.” (P10)  “I lived with my family…and I was going through this. I think they kind of thought I was just kind of milking it, you know, because my head is fine. I can still laugh, you know, I’m not gonna be, but it was kind of, you know, I remember their faces when I told them the results and they were just shocked, you know. So, but it was I think it helped even emotionally it helped, you know, because you know you’re not going crazy.” (P6). |
| **Medical care seeking – Allied HCPs** | All patients had visited allied health care providers (primarily physiotherapists, massage therapists, and chiropractors for LBP treatment (N=10) | “I have been doing a lot of physio.” (P2)  “And right now, I’m seeing another massage therapist who thinks that he can help yeah.” (P7)  "And, you know, I spent thousands and thousands of dollars going to different chiropractors.” (P5) |
|  | Allied HCPs recommended stretches or exercises for them to complete at home. (N=3) | “And when I went there (massage therapist) they did explain some moves that I can keep doing for a while, so that there’s less pressure on my bones and my muscles are stronger.” (P1).  “She (physiotherapist) gave me some exercises and a lot of stretching. Just different ones I have never seen before and it worked.” (P3) |
|  | Some patients were pleased with the care received from allied HCPs, others were not. (N=6) | “I felt a lot better like almost immediately when she (chiropractor)…she cracked my back, I could just feel the relief that it was pretty nice.” (P10).  “I wish I could see my massage therapist more than just like once a week because it does help and I think that if I saw them more often, it would help it even more.” (P3).  “And I thought the physiotherapist helped just increase, and it was so, such a small, small increase, increase in ability a little bit. But it was very slow.” (P3).  “I did physiotherapy, which I don't do anymore 'cause that didn't help me at all.” (P4).  “But like I said I haven’t been overly successful and like sometimes I wondering if like the physio is actually helping or is it just like time?” (P2).  “I’ve tried massage therapy which is awful for me.” (P6). |
|  | Some patients were frustrated with limitations placed on their ability to use allied healthcare because of the cost involved. (N= 2) | “I really feel like if I kept going (to chiropractor) I would have been in a much better spot but it’s just so expensive I just can’t keep up with it.” (P10). |
