# Supplementary material for: An exploration of patients’ perceptions and coping strategies for LBP
Source: PLoS One. 2025 Jun 9;20(6):e0324859. doi: 10.1371/journal.pone.0324859 (PMC12148076; doi:10.1371/journal.pone.0324859)
Supplement: Attachment — Submitted filename: Response to reviews.docx [file pone.0324859.s004.docx]

**We would like to thank all the reviewers for their thoughtful input on our manuscript. We have responded to each of their comments below.**

**Reviewer #1:**

- Enhancing clarity in the results section, perhaps with visual summaries or subheadings, would improve readability.

Thank you. We have included 3 levels of subheadings in our results section according to Plos One author guidelines to enhance clarity.

- The authors may wish to discuss how regional factors, such as Newfoundland and Labrador’s healthcare system structure and local patient attitudes, might further inform the interpretation of these findings. Differences in healthcare access, especially in rural areas, could influence patients' strong preference for imaging and perceptions of care utility, as well as shape their expectations for diagnosis and management. Additionally, cultural factors specific to NL may impact patients' beliefs about LBP and their reliance on community-shared experiences, potentially reinforcing particular attitudes toward imaging and care-seeking behaviors. Discussing these regional aspects (if existing) would provide a more nuanced understanding of patient expectations and could inform regionally tailored interventions in LBP management.

Thank you for this thoughtful comment. At this time, we don’t have knowledge of potential regional differences in patient attitudes and/or healthcare access or cultural differences that might influence patient perceptions of their LBP and/or their expectations for treatment. Explorations of these types of differences was beyond the scope of the current study. As such, we have added text to our limitations section to acknowledge how these differences (if existing) could have provided a more nuanced understanding of patient expectations to inform regionally tailored interventions in LBP management.

- Addressing formal data saturation assessment would also strengthen the methodological rigor.

Thank you for this comment. We agree that a formal assessment of data saturation strengthens our study. To this end, we have completed a formal assessment of thematic saturation per the method proposed by Guest et al. 2020 (Guest G, Namey E, Chen M (2020) A simple method to assess and report thematic saturation in qualitative research. PLOS ONE 15(5): e0232076. https://doi.org/10.1371/journal.pone.0232076).

This analysis showed that saturation was achieved after eight interviews – the point at which no new themes had emerged from the data. We have added this to our results section and removed lines from our limitations regarding the limitation of not formally assessing saturation.

**Reviewer #2:**

- I am concerned about the old/outdated references where there are more recent studies available. There are only 11 references from the last 5 years, 2 from the last ten years and 16 older than 10 years - and this is a field where research on LBP and contextual factors in PAIN has exploded in the last five years

Thank you for this helpful comment. We have updated our manuscript (primarily the intro and discussion). The reference list now includes 18 references from the last five years, 7 from the last 10 years, and 10 older than 10 years.

- As this is a study that has been done in many other countries, I feel that discussing similar and contrasting findings is worthwhile.

Thank you for raising this issue. In our discussion section we have included a subsection called “comparision with the literature” that discusses the similarities and differences between our findings and the wider literature. Rather than cite all the individual studies on these topics we have primarily cited systematic and/or scoping reviews in our comparisons as these include data from relevant studies conducted around the world. For example, the Lim systematic review included 41 studies conducted in 13 countries, the Gron scoping review included 81 studies conducted in 39 countries, and the Verbeek systematic review included 20 studies conducted in 7 countries. We have added text to this section of our manuscript to enhance clarity on this issue.

- Also- the reason for duplicating the study in your community/state is not clear.

Thank you for this comment. This investigation was completed as part of a larger project to develop an intervention to improve overall guideline management of back pain, particularly around recommendations for imaging. While many studies exist about why patients seek care, there isn’t a lot of qualitative literature asking patients directly about if and **why** they want imaging as part of their care. We have adjusted the text of the introduction to make this more clear.

- What are the implications for your findings? Is it going to change current practice and how can your study contribute to that?

Thank you for this comment. These issues have been addressed in the section Implications for Research and Practice in our Discussion section but we have highlighted key implications here for the reviewer’s benefit. The implications of our findings are largely related to education for patients and a mismatch between physician and patient views about what patients need and want from their encounters when experiencing LBP. Our findings suggest that practice should change particularly around improving patient education related to diagnosis, prognosis, and imaging as well as patient-provider communication about these topics. The results of our study indicate that information most important to patients is not being adequately communicated to patients and that patients are misinformed about the usefulness of imaging.

**Reviewer #3:**

- In this article the researchers interviewed ten patients with low back pain and had recently visited a family practice physician. Presumably this included some chronic and acute pain as it was not clear if they excluded acute low back pain.

Thank you for raising this point. We included participants who had visited their family doctor for LBP in the past year and did not exclude based on whether they were experiencing acute or chronic pain. Only those with a specific cause were excluded from participation. We have clarified this in the method section.

- I think the sample is on the smaller side and the lack of rigorous inclusion/exclusion criteria allowed for more heterogeneity in the sample limiting in some ways the generalization of findings to a specific group of people e.g., chronic low back pain.

The purpose of our study was to understand why all patients with LBP visit their family doctors and if and why they are looking for imaging. It is our hope that this information will help to inform an educational intervention for use in primary care with all back pain patients. However, we acknowledge that it is possible that these groups have difference reasons for visiting their doctors and have different desires for imaging. We have added text acknowledging this to the implications for research and practice section of the discussion.

- The relationship between the patient perceptions and the low back guidelines, especially around imaging, needs to be a little bit more nuanced as this study is only capturing one perspective (that of patients). Also, I feel that the authors should also more explicitly state what they are using as their guidelines for comparison. I agree that patient beliefs, emotions, and coping strategies may create barriers for adherence to guidelines or create situations where the patient’s expectation of care is not met. However, in some cases, e.g., no response to treatment, imaging might be indicated and then of value, if only to rule out serious pathology. I think this point could have been better emphasized and I suggest that the authors avoid calling imaging “low-value” writ large. Below are some specific comments.

Thank you for these comments. We have addressed them in the specific points noted below.

Abstract

Line 35, spell out NL, international audiences may not be familiar with this abbreviation.

Done

Introduction

Ln 63, I think it is important to concisely provide guidelines for LBP, maybe a table would be helpful.

We have added text to the introduction summarizing LBP guidelines, particularly related to imaging, and provided updated references.

Ln 64, the parentheses in this line is unclosed, i.e., missing “)”

Fixed – removed the bracket.

Ln 83, I suggest changing “illness” to “symptoms” since illness is more associated with a organic biological cause, which is not necessarily applicable with pain.

Adjusted as requested.

Ln 86, Remove “(refs)”

Adjusted as requested.

Ln 87, I would state instead of “becoming overprotective…” this can “upregulate protective pain behaviors and increase avoidance of valued activities and movement, thereby creating barriers to adherence to guideline-concordant care.”

Adjusted as requested.

Ln 89-91, “To date..” I would remove this sentence as it is not directly relevant to the rest of the writing in the paragraph. Your study is not evaluating causality, and I think that causality is a challenging to understand with how beliefs are related to behavior. So best not to open that can of worms here.

Removed as requested.

**Note to self – this removed a reference so they will have to be updated.**

Ln 99, define NL

Adjusted as requested

Method

Ln 117, capitalize “r” in regulation

Adjusted as requested

Ln 148, define “CAD”

Completed as requested.

Discussion

Ln 471, “low value” I would change this to “use” so as not to sound like you are making the blanketed statement that imaging is low value. Obviously, there are some cases where it is indicated, if only to rule out serious pathologies.

Adjusted as requested.

Ln 479, citation is needed for this statement. Additionally, “The ACR accordingly recommends imaging for low back pain in (1) patients who have had up to 6 weeks of medical management and physical therapy for low back pain without improvement in symptoms, and (2) patients with red flag symptoms for serious pathology.1”

1.Patel ND, Broderick DF, Burns J, et al. ACR appropriateness criteria low back pain. J Am Coll Radiol. 2016. September;13(9):1069-1078. doi: 10.1016/j.jacr.2016.06.008. [DOI] [PubMed] [Google Scholar]

I think this is an important point. That it is indicated for imaging if there is no improvement with conservative treatment. Chances are many patients, especially those in this cohort, have likely not had improvement and therefore imaging would be indicated. Imaging in this case can be used to help reaffirm the complex aspects of pain that are not biomedical (if there is no serious pathology) and if imaging is not given after no response to treatment this could be a legitimate source of frustration with patients.

Thank you for raising this concern as it highlights an interesting point. The guidelines around imaging for patients based on pain duration and management outcomes are actually too vague and are often interpreted as you have suggested. However, the advice for a patient who has had pain for 6 weeks or more, and failed conservative treatment, is to reassess the patient and, again, image only if red flags are suspected. In the reference you suggest, I think you are referring to the imaging recommendation for variant 3 as I have copied below. The reason for imaging at that point is still to rule out suspected red flags or for a surgeon who may be considering the patient as a candidate for surgery. This mismatch between the guideline recommendation and its interpretation for practice to be used as you suggested is widespread and something that should be clarified. In fact, using imaging to reassure a patient that no specific cause for their pain has come up so many times in the qualitative literature. We are currently planning a prospective cohort study to better understand how often imaging is being used for that exact reason to provide guideline producers with suggestions for clarification. As such we haven't adjusted the text in this manuscript regarding this guideline recommendation but certainly sympathize with this reason for imaging use.

“Variant 3 - Subacute or chronic low back pain with or without radiculopathy. Surgery or intervention candidate with persistent or progressive symptoms during or following 6 weeks of optimal medical management. Initial imaging.

Ln 503, suggest removing “these low-value” and changing “images” to “imaging”

Adjusted as requested.

Ln 508, again remove-“low value”

Removed as requested.

Ln 502-509, it is important to realize there are two motivations for reducing imaging (1) to control costs of healthcare and (2) reduce iatrogenic effects of stereotyped threat (e.g., attributing pain to normal age related changes). Controlling cost is often not a patient-centered goal, it is a healthcare centered goal, since it is about containing cost. I believe it is natural that when the healthcare system and the patient lack alignment in goals frustration and other emotions are likely to occur. With respect to the second point, improving communication and expectations of imaging may allow the use of imaging (when appropriate) while also minimizing harms. In short I think this point is nuanced here and I think you might be careful not to sound like you are blaming patients for seeking imaging, when in fact in some cases it may be warranted, and also it is natural to want more information for an unexplained or not well understood health condition.

Thank you for this comment. While it was not our intention, we understand how we may have sounded as if we were blaming patients for seeking imaging. We have revised this section and added some additional text in an effort to improve the clarity of our message. Essentially, we meant to point out that physicians report that they sometimes image outside the guidelines because they believe that imaging is what their patients really want from their visit. But the patients in our study told us that what they really want is more information about their LBP – a diagnosis, information about prognosis and potential treatments. We also added a point about the need for researchers to assess the prevalence of imaging outside the guidelines at patients’ requests in order to gain an understanding of the magnitude of the problem. This will help researchers to develop targeted interventions to improve management of LBP.
