# Supplementary material for: An exploration of patients’ perceptions and coping strategies for LBP
Source: PLoS One. 2025 Jun 9;20(6):e0324859. doi: 10.1371/journal.pone.0324859 (PMC12148076; doi:10.1371/journal.pone.0324859)
Supplement: Peer review history

# Author comment: Author response to Decision Letter 0

**DOI:** 10.1371/journal.pone.0324859.r001

28 Aug 2024

# Peer review: Decision Letter 0

**DOI:** 10.1371/journal.pone.0324859.r002

**Authors:** Tadashi Ito

9 Mar 2025

PONE-D-24-37525An exploration of patients’ perceptions and coping strategies for low back painPLOS ONE

Dear Dr. Pike, 

Thank you for submitting your manuscript to PLOS ONE. After careful consideration, we feel that it has merit but does not fully meet PLOS ONE’s publication criteria as it currently stands. Therefore, we invite you to submit a revised version of the manuscript that addresses the points raised during the review process.

Please submit your revised manuscript by Apr 23 2025 11:59PM. If you will need more time than this to complete your revisions, please reply to this message or contact the journal office at plosone@plos.org . When you're ready to submit your revision, log on to https://www.editorialmanager.com/pone/ and select the 'Submissions Needing Revision' folder to locate your manuscript file.

Please include the following items when submitting your revised manuscript:

- A rebuttal letter that responds to each point raised by the academic editor and reviewer(s). You should upload this letter as a separate file labeled 'Response to Reviewers'.
- A marked-up copy of your manuscript that highlights changes made to the original version. You should upload this as a separate file labeled 'Revised Manuscript with Track Changes'.
- An unmarked version of your revised paper without tracked changes. You should upload this as a separate file labeled 'Manuscript'.

If you would like to make changes to your financial disclosure, please include your updated statement in your cover letter. Guidelines for resubmitting your figure files are available below the reviewer comments at the end of this letter.

If applicable, we recommend that you deposit your laboratory protocols in protocols.io to enhance the reproducibility of your results. Protocols.io assigns your protocol its own identifier (DOI) so that it can be cited independently in the future. For instructions see: https://journals.plos.org/plosone/s/submission-guidelines#loc-laboratory-protocols . Additionally, PLOS ONE offers an option for publishing peer-reviewed Lab Protocol articles, which describe protocols hosted on protocols.io. Read more information on sharing protocols at https://plos.org/protocols?utm_medium=editorial-email&utm_source=authorletters&utm_campaign=protocols .

We look forward to receiving your revised manuscript.

Kind regards,

Tadashi Ito

Academic Editor

PLOS ONE

Journal Requirements:

When submitting your revision, we need you to address these additional requirements.

1. Please ensure that your manuscript meets PLOS ONE's style requirements, including those for file naming. The PLOS ONE style templates can be found at

https://journals.plos.org/plosone/s/file?id=wjVg/PLOSOne_formatting_sample_main_body.pdf and

https://journals.plos.org/plosone/s/file?id=ba62/PLOSOne_formatting_sample_title_authors_affiliations.pdf

2. Please include captions for your Supporting Information files at the end of your manuscript, and update any in-text citations to match accordingly. Please see our Supporting Information guidelines for more information: http://journals.plos.org/plosone/s/supporting-information .

3. Please review your reference list to ensure that it is complete and correct. If you have cited papers that have been retracted, please include the rationale for doing so in the manuscript text, or remove these references and replace them with relevant current references. Any changes to the reference list should be mentioned in the rebuttal letter that accompanies your revised manuscript. If you need to cite a retracted article, indicate the article’s retracted status in the References list and also include a citation and full reference for the retraction notice.

Additional Editor Comments :

A thorough review of the reviewers' comments is imperative to ensure the accuracy and thoroughness of the text.

Reviewers' comments:

Reviewer's Responses to Questions

**Comments to the Author**

1. Is the manuscript technically sound, and do the data support the conclusions?

The manuscript must describe a technically sound piece of scientific research with data that supports the conclusions. Experiments must have been conducted rigorously, with appropriate controls, replication, and sample sizes. The conclusions must be drawn appropriately based on the data presented. 

Reviewer #1: Yes

Reviewer #2: Yes

Reviewer #3: Yes

**********

2. Has the statistical analysis been performed appropriately and rigorously? 

Reviewer #1: N/A

Reviewer #2: I Don't Know

Reviewer #3: Yes

**********

3. Have the authors made all data underlying the findings in their manuscript fully available?

The PLOS Data policy requires authors to make all data underlying the findings described in their manuscript fully available without restriction, with rare exception (please refer to the Data Availability Statement in the manuscript PDF file). The data should be provided as part of the manuscript or its supporting information, or deposited to a public repository. For example, in addition to summary statistics, the data points behind means, medians and variance measures should be available. If there are restrictions on publicly sharing data—e.g. participant privacy or use of data from a third party—those must be specified.

Reviewer #1: Yes

Reviewer #2: Yes

Reviewer #3: No

**********

4. Is the manuscript presented in an intelligible fashion and written in standard English?

PLOS ONE does not copyedit accepted manuscripts, so the language in submitted articles must be clear, correct, and unambiguous. Any typographical or grammatical errors should be corrected at revision, so please note any specific errors here.

Reviewer #1: Yes

Reviewer #2: Yes

Reviewer #3: Yes

**********

5. Review Comments to the Author

Please use the space provided to explain your answers to the questions above. You may also include additional comments for the author, including concerns about dual publication, research ethics, or publication ethics. (Please upload your review as an attachment if it exceeds 20,000 characters)

Reviewer #1: The manuscript offers valuable insights into patient perceptions and coping strategies for LBP, highlighting important discrepancies between patient expectations and clinical practices. The use of the CSM framework to categorize patient beliefs and coping mechanisms is a strength, and the findings have clear implications for improving patient-provider communication and reducing low-value imaging. Enhancing clarity in the results section, perhaps with visual summaries or subheadings, would improve readability. The authors may wish to discuss how regional factors, such as Newfoundland and Labrador’s healthcare system structure and local patient attitudes, might further inform the interpretation of these findings. Differences in healthcare access, especially in rural areas, could influence patients' strong preference for imaging and perceptions of care utility, as well as shape their expectations for diagnosis and management. Additionally, cultural factors specific to NL may impact patients' beliefs about LBP and their reliance on community-shared experiences, potentially reinforcing particular attitudes toward imaging and care-seeking behaviors. Discussing these regional aspects (if existing) would provide a more nuanced understanding of patient expectations and could inform regionally tailored interventions in LBP management. Addressing formal data saturation assessment would also strengthen the methodological rigor. Overall, this study contributes important data to the field and highlights areas where healthcare practices may better align with patient needs.

Reviewer #2: Thanks for a well-written report

I am concerned about the old/outdated referenceds where there are more recent studies avaialble

There are only 11 references from the last 5 years, 2 from the last ten years and 16 older than 10 years - and this is a field where research on LBP and contextual factors in PAIN has exploded in the last five years

Oliveira CB, Maher CG, Pinto RZ, Traeger AC, Lin CW, Chenot JF, Van Tulder M, Koes BW. Clinical practice guidelines for the management of non-specific low back pain in primary care: an updated overview. European Spine Journal. 2018 Nov;27:2791-803.

Kreiner DS, Matz P, Bono CM, Cho CH, Easa JE, Ghiselli G, Ghogawala Z, Reitman CA, Resnick DK, Watters III WC, Annaswamy TM. Guideline summary review: an evidence-based clinical guideline for the diagnosis and treatment of low back pain. The Spine Journal. 2020 Jul 1;20(7):998-1024.

Meroni R, Piscitelli D, Ravasio C, Vanti C, Bertozzi L, De Vito G, Perin C, Guccione AA, Cerri CG, Pillastrini P. Evidence for managing chronic low back pain in primary care: a review of recommendations from high-quality clinical practice guidelines. Disability and rehabilitation. 2021 Mar 27;43(7):1029-43.

Corp N, Mansell G, Stynes S, Wynne‐Jones G, Morsø L, Hill JC, van der Windt DA. Evidence‐based treatment recommendations for neck and low back pain across Europe: a systematic review of guidelines. European Journal of Pain. 2021 Feb;25(2):275-95.

George SZ, Fritz JM, Silfies SP, Schneider MJ, Beneciuk JM, Lentz TA, Gilliam JR, Hendren S, Norman KS, Beattie PF, Bishop MD. Interventions for the management of acute and chronic low back pain: revision 2021: clinical practice guidelines linked to the international classification of functioning, disability and health from the academy of orthopaedic physical therapy of the American Physical Therapy Association. Journal of Orthopaedic & Sports Physical Therapy. 2021 Nov;51(11):CPG1-60.

As this is a study that has been doen in many other countries I feel that discussing similar and contrasting findings is worthwhile

Also- the reason for duplicating the study in yur community/state is not clear. What are the imlications for your findings? Is it going to change current practice and how can your study contribute to that?

Reviewer #3: In this article the researchers interviewed ten patients with low back pain and had recently visited a family practice physician. Presumably this included some chronic and acute pain as it was not clear if they excluded acute low back pain. The authors were guided by the commonsense model of self-regulation and they used this in developing a semi structured interview guide. The authors framed their findings in juxtaposition to management guidelines for chronic low back pain.

Overall, I found the manuscript generally well written and easy to follow. I think the sample is on the smaller side and the lack of rigorous inclusion/exclusion criteria allowed for more heterogeneity in the sample limiting in some ways the generalization of findings to a specific group of people e.g., chronic low back pain. I didn’t find overall much of the findings incredibly novel since previous studies have reported similar findings. The relationship between the patient perceptions and the low back guidelines, especially around imaging, needs to be a little bit more nuanced as this study is only capturing one perspective (that of patients). Also, I feel that the authors should also more explicitly state what they are using as their guidelines for comparison. I agree that patient beliefs, emotions, and coping strategies may create barriers for adherence to guidelines or create situations where the patient’s expectation of care is not met. However, in some cases, e.g., no response to treatment, imaging might be indicated and then of value, if only to rule out serious pathology. I think this point could have been better emphasized and I suggest that the authors avoid calling imaging “low-value” writ large. Below are some specific comments.

Abstract

Line 35, spell out NL, international audiences may not be familiar with this abbreviation.

Introduction

Ln 63, I think it is important to concisely provide guidelines for LBP, maybe a table would be helpful.

Ln 64, the parentheses in this line is unclosed, i.e., missing “)”

Ln 83, I suggest changing “illness” to “symptoms” since illness is more associated with a organic biological cause, which is not necessarily applicable with pain.

Ln 86, Remove “(refs)”

Ln 87, I would state instead of “becoming overprotective…” this can “upregulate protective pain behaviors and increase avoidance of valued activities and movement, thereby creating barriers to adherence to guideline-concordant care.”

Ln 89-91, “To date..” I would remove this sentence as it is not directly relevant to the rest of the writing in the paragraph. Your study is not evaluating causality, and I think that causality is a challenging to understand with how beliefs are related to behavior. So best not to open that can of worms here.

Ln 99, define NL

Method

Ln 117, capitalize “r” in regulation

Ln 148, define “CAD”

Discussion

Ln 471, “low value” I would change this to “use” so as not to sound like you are making the blanketed statement that imaging is low value. Obviously, there are some cases where it is indicated, if only to rule out serious pathologies.

Ln 479, citation is needed for this statement. Additionally, “The ACR accordingly recommends imaging for low back pain in (1) patients who have had up to 6 weeks of medical management and physical therapy for low back pain without improvement in symptoms, and (2) patients with red flag symptoms for serious pathology.1”

1.Patel ND, Broderick DF, Burns J, et al. ACR appropriateness criteria low back pain. J Am Coll Radiol. 2016. September;13(9):1069-1078. doi: 10.1016/j.jacr.2016.06.008. [DOI] [PubMed] [Google Scholar]

I think this is an important point. That it is indicated for imaging if there is no improvement with conservative treatment. Chances are many patients, especially those in this cohort, have likely not had improvement and therefore imaging would be indicated. Imaging in this case can be used to help reaffirm the complex aspects of pain that are not biomedical (if there is no serious pathology) and if imaging is not given after no response to treatment this could be a legitimate source of frustration with patients.

Ln 503, suggest removing “these low-value” and changing “images” to “imaging”

Ln 508, again remove-“low value”

Ln 502-509, it is important to realize there are two motivations for reducing imaging (1) to control costs of healthcare and (2) reduce iatrogenic effects of stereotyped threat (e.g., attributing pain to normal age related changes). Controlling cost is often not a patient-centered goal, it is a healthcare centered goal, since it is about containing cost. I believe it is natural that when the healthcare system and the patient lack alignment in goals frustration and other emotions are likely to occur. With respect to the second point, improving communication and expectations of imaging may allow the use of imaging (when appropriate) while also minimizing harms. In short I think this point is nuanced here and I think you might be careful not to sound like you are blaming patients for seeking imaging, when in fact in some cases it may be warranted, and also it is natural to want more information for an unexplained or not well understood health condition.

**********

6. PLOS authors have the option to publish the peer review history of their article (what does this mean? ). If published, this will include your full peer review and any attached files.

If you choose “no”, your identity will remain anonymous but your review may still be made public.

**Do you want your identity to be public for this peer review?** For information about this choice, including consent withdrawal, please see our Privacy Policy .

Reviewer #1: No

Reviewer #2: **Yes: ** Ina Diener

Reviewer #3: No

**********

[NOTE: If reviewer comments were submitted as an attachment file, they will be attached to this email and accessible via the submission site. Please log into your account, locate the manuscript record, and check for the action link "View Attachments". If this link does not appear, there are no attachment files.]

While revising your submission, please upload your figure files to the Preflight Analysis and Conversion Engine (PACE) digital diagnostic tool, https://pacev2.apexcovantage.com/ . PACE helps ensure that figures meet PLOS requirements. To use PACE, you must first register as a user. Registration is free. Then, login and navigate to the UPLOAD tab, where you will find detailed instructions on how to use the tool. If you encounter any issues or have any questions when using PACE, please email PLOS at figures@plos.org . Please note that Supporting Information files do not need this step.

# Author comment: Author response to Decision Letter 1

**DOI:** 10.1371/journal.pone.0324859.r003

14 Apr 2025

We would like to thank all the reviewers for their thoughtful input on our manuscript. We have responded to each of their comments below.

Reviewer #1:

• Enhancing clarity in the results section, perhaps with visual summaries or subheadings, would improve readability.

Thank you. We have included 3 levels of subheadings in our results section according to Plos One author guidelines to enhance clarity.

• The authors may wish to discuss how regional factors, such as Newfoundland and Labrador’s healthcare system structure and local patient attitudes, might further inform the interpretation of these findings. Differences in healthcare access, especially in rural areas, could influence patients' strong preference for imaging and perceptions of care utility, as well as shape their expectations for diagnosis and management. Additionally, cultural factors specific to NL may impact patients' beliefs about LBP and their reliance on community-shared experiences, potentially reinforcing particular attitudes toward imaging and care-seeking behaviors. Discussing these regional aspects (if existing) would provide a more nuanced understanding of patient expectations and could inform regionally tailored interventions in LBP management.

Thank you for this thoughtful comment. At this time, we don’t have knowledge of potential regional differences in patient attitudes and/or healthcare access or cultural differences that might influence patient perceptions of their LBP and/or their expectations for treatment. Explorations of these types of differences was beyond the scope of the current study. As such, we have added text to our limitations section to acknowledge how these differences (if existing) could have provided a more nuanced understanding of patient expectations to inform regionally tailored interventions in LBP management.

• Addressing formal data saturation assessment would also strengthen the methodological rigor.

Thank you for this comment. We agree that a formal assessment of data saturation strengthens our study. To this end, we have completed a formal assessment of thematic saturation per the method proposed by Guest et al. 2020 (Guest G, Namey E, Chen M (2020) A simple method to assess and report thematic saturation in qualitative research. PLOS ONE 15(5): e0232076. https://doi.org/10.1371/journal.pone.0232076).

This analysis showed that saturation was achieved after eight interviews – the point at which no new themes had emerged from the data. We have added this to our results section and removed lines from our limitations regarding the limitation of not formally assessing saturation.

Reviewer #2:

• I am concerned about the old/outdated references where there are more recent studies available. There are only 11 references from the last 5 years, 2 from the last ten years and 16 older than 10 years - and this is a field where research on LBP and contextual factors in PAIN has exploded in the last five years

Thank you for this helpful comment. We have updated our manuscript (primarily the intro and discussion). The reference list now includes 18 references from the last five years, 7 from the last 10 years, and 10 older than 10 years.

• As this is a study that has been done in many other countries, I feel that discussing similar and contrasting findings is worthwhile.

Thank you for raising this issue. In our discussion section we have included a subsection called “comparision with the literature” that discusses the similarities and differences between our findings and the wider literature. Rather than cite all the individual studies on these topics we have primarily cited systematic and/or scoping reviews in our comparisons as these include data from relevant studies conducted around the world. For example, the Lim systematic review included 41 studies conducted in 13 countries, the Gron scoping review included 81 studies conducted in 39 countries, and the Verbeek systematic review included 20 studies conducted in 7 countries. We have added text to this section of our manuscript to enhance clarity on this issue.

• Also- the reason for duplicating the study in your community/state is not clear.

Thank you for this comment. This investigation was completed as part of a larger project to develop an intervention to improve overall guideline management of back pain, particularly around recommendations for imaging. While many studies exist about why patients seek care, there isn’t a lot of qualitative literature asking patients directly about if and why they want imaging as part of their care. We have adjusted the text of the introduction to make this more clear.

• What are the implications for your findings? Is it going to change current practice and how can your study contribute to that?

Thank you for this comment. These issues have been addressed in the section Implications for Research and Practice in our Discussion section but we have highlighted key implications here for the reviewer’s benefit. The implications of our findings are largely related to education for patients and a mismatch between physician and patient views about what patients need and want from their encounters when experiencing LBP. Our findings suggest that practice should change particularly around improving patient education related to diagnosis, prognosis, and imaging as well as patient-provider communication about these topics. The results of our study indicate that information most important to patients is not being adequately communicated to patients and that patients are misinformed about the usefulness of imaging.

Reviewer #3:

• In this article the researchers interviewed ten patients with low back pain and had recently visited a family practice physician. Presumably this included some chronic and acute pain as it was not clear if they excluded acute low back pain.

Thank you for raising this point. We included participants who had visited their family doctor for LBP in the past year and did not exclude based on whether they were experiencing acute or chronic pain. Only those with a specific cause were excluded from participation. We have clarified this in the method section.

• I think the sample is on the smaller side and the lack of rigorous inclusion/exclusion criteria allowed for more heterogeneity in the sample limiting in some ways the generalization of findings to a specific group of people e.g., chronic low back pain.

The purpose of our study was to understand why all patients with LBP visit their family doctors and if and why they are looking for imaging. It is our hope that this information will help to inform an educational intervention for use in primary care with all back pain patients. However, we acknowledge that it is possible that these groups have difference reasons for visiting their doctors and have different desires for imaging. We have added text acknowledging this to the implications for research and practice section of the discussion.

• The relationship between the patient perceptions and the low back guidelines, especially around imaging, needs to be a little bit more nuanced as this study is only capturing one perspective (that of patients). Also, I feel that the authors should also more explicitly state what they are using as their guidelines for comparison. I agree that patient beliefs, emotions, and coping strategies may create barriers for adherence to guidelines or create situations where the patient’s expectation of care is not met. However, in some cases, e.g., no response to treatment, imaging might be indicated and then of value, if only to rule out serious pathology. I think this point could have been better emphasized and I suggest that the authors avoid calling imaging “low-value” writ large. Below are some specific comments.

Thank you for these comments. We have addressed them in the specific points noted below.

Abstract

Line 35, spell out NL, international audiences may not be familiar with this abbreviation.

Done

Introduction

Ln 63, I think it is important to concisely provide guidelines for LBP, maybe a table would be helpful.

We have added text to the introduction summarizing LBP guidelines, particularly related to imaging, and provided updated references.

Ln 64, the parentheses in this line is unclosed, i.e., missing “)”

Fixed – removed the bracket.

Ln 83, I suggest changing “illness” to “symptoms” since illness is more associated with a organic biological cause, which is not necessarily applicable with pain.

Adjusted as requested.

Ln 86, Remove “(refs)”

Adjusted as requested.

Ln 87, I would state instead of “becoming overprotective…” this can “upregulate protective pain behaviors and increase avoidance of valued activities and movement, thereby creating barriers to adherence to guideline-concordant care.”

Adjusted as requested.

Ln 89-91, “To date..” I would remove this sentence as it is not directly relevant to the rest of the writing in the paragraph. Your study is not evaluating causality, and I think that causality is a challenging to understand with how beliefs are related to behavior. So best not to open that can of worms here.

Removed as requested.

Note to self – this removed a reference so they will have to be updated.

Ln 99, define NL

Adjusted as requested

Method

Ln 117, capitalize “r” in regulation

Adjusted as requested

Ln 148, define “CAD”

Completed as requested.

Discussion

Ln 471, “low value” I would change this to “use” so as not to sound like you are making the blanketed statement that imaging is low value. Obviously, there are some cases where it is indicated, if only to rule out serious pathologies.

Adjusted as requested.

Ln 479, citation is needed for this statement. Additionally, “The ACR accordingly recommends imaging for low back pain in (1) patients who have had up to 6 weeks of medical management and physical therapy for low back pain without improvement in symptoms, and (2) patients with red flag symptoms for serious pathology.1”

1.Patel ND, Broderick DF, Burns J, et al. ACR appropriateness criteria low back pain. J Am Coll Radiol. 2016. September;13(9):1069-1078. doi: 10.1016/j.jacr.2016.06.008. [DOI] [PubMed] [Google Scholar]

I think this is an important point. That it is indicated for imaging if there is no improvement with conservative treatment. Chances are many patients, especially those in this cohort, have likely not had improvement and therefore imaging would be indicated. Imaging in this case can be used to help reaffirm the complex aspects of pain that are not biomedical (if there is no serious pathology) and if imaging is not given after no response to treatment this could be a legitimate source of frustration with patients.

Thank you for raising this concern as it highlights an interesting point. The guidelines around imaging for patients based on pain duration and management outcomes are actually too vague and are often interpreted as you have suggested. However, the advice for a patient who has had pain for 6 weeks or more, and failed conservative treatment, is to reassess the patient and, again, image only if red flags are suspected. In the reference you suggest, I think you are referring to the imaging recommendation for variant 3 as I have copied below. The reason for imaging at that point is still to rule out suspected red flags or for a surgeon who may be considering the patient as a candidate for surgery. This mismatch between the guideline recommendation and its interpretation for practice to be used as you suggested is widespread and something that should be clarified. In fact, using imaging to reassure a patient that no specific cause for their pain has come up so many times in the qualitative literature. We are currently planning a prospective cohort study to better understand how often imaging is being used for that exact reason to provide guideline producers with suggestions for clarification. As such we haven't adjusted the text in this manuscript regarding this guideline recommendation but certainly sympathize with this reason for imaging use.

“Variant 3 - Subacute or chronic low back pain with or without radiculopathy. Surgery or intervention candidate with persistent or progressive symptoms during or following 6 weeks of optimal medical management. Initial imaging.”

Ln 503, suggest removing “these low-value” and changing “images” to “imaging”

Adjusted as requested.

Ln 508, again remove-“low value”

Removed as requested.

Ln 502-509, it is important to realize there are two motivations for reducing imaging (1) to control costs of healthcare and (2) reduce iatrogenic effects of stereotyped threat (e.g., attributing pain to normal age related changes). Controlling cost is often not a patient-centered goal, it is a healthcare centered goal, since it is about containing cost. I believe it is natural that when the healthcare system and the patient lack alignment in goals frustration and other emotions are likely to occur. With respect to the second point, improving communication and expectations of imaging may allow the use of imaging (when appropriate) while also minimizing harms. In short I think this point is nuanced here and I think you might be careful not to sound like you are blaming patients for seeking imaging, when in fact in some cases it may be warranted, and also it is natural to want more information for an unexplained or not well understood health condition.

Thank you for this comment. While it was not our intention, we understand how we may have sounded as if we were blaming patients for seeking imaging. We have revised this section and added some additional text in an effort to improve the clarity of our message. Essentially, we meant to point out that physicians report that they sometimes image outside the guidelines because they believe that imaging is what their patients really want from their visit. But the patients in our study told us that what they really want is more information about their LBP – a diagnosis, information about prognosis and potential treatments. We also added a point about the need for researchers to assess the prevalence of imaging outside the guidelines at patients’ requests in order to gain an understanding of the magnitude of the problem. This will help researchers to develop targeted interventions to improve management of LBP.

Attachment Submitted filename: Response to reviews.docx

# Peer review: Decision Letter 1

**DOI:** 10.1371/journal.pone.0324859.r004

**Authors:** Tadashi Ito

2 May 2025

An exploration of patients’ perceptions and coping strategies for low back pain

PONE-D-24-37525R1

Dear Dr. Andrea Pike,

We’re pleased to inform you that your manuscript has been judged scientifically suitable for publication and will be formally accepted for publication once it meets all outstanding technical requirements.

Within one week, you’ll receive an e-mail detailing the required amendments. When these have been addressed, you’ll receive a formal acceptance letter and your manuscript will be scheduled for publication.

An invoice will be generated when your article is formally accepted. Please note, if your institution has a publishing partnership with PLOS and your article meets the relevant criteria, all or part of your publication costs will be covered. Please make sure your user information is up-to-date by logging into Editorial Manager at Editorial Manager®  and clicking the ‘Update My Information' link at the top of the page. If you have any questions relating to publication charges, please contact our Author Billing department directly at authorbilling@plos.org.

If your institution or institutions have a press office, please notify them about your upcoming paper to help maximize its impact. If they’ll be preparing press materials, please inform our press team as soon as possible -- no later than 48 hours after receiving the formal acceptance. Your manuscript will remain under strict press embargo until 2 pm Eastern Time on the date of publication. For more information, please contact onepress@plos.org.

Kind regards,

Tadashi Ito

Academic Editor

PLOS ONE

Additional Editor Comments (optional):

The necessary corrections were duly implemented. The present manuscript has been accepted for publication.

Reviewers' comments:

Reviewer's Responses to Questions

**Comments to the Author**

1. If the authors have adequately addressed your comments raised in a previous round of review and you feel that this manuscript is now acceptable for publication, you may indicate that here to bypass the “Comments to the Author” section, enter your conflict of interest statement in the “Confidential to Editor” section, and submit your "Accept" recommendation.

Reviewer #1: All comments have been addressed

Reviewer #2: All comments have been addressed

**********

2. Is the manuscript technically sound, and do the data support the conclusions?

The manuscript must describe a technically sound piece of scientific research with data that supports the conclusions. Experiments must have been conducted rigorously, with appropriate controls, replication, and sample sizes. The conclusions must be drawn appropriately based on the data presented. 

Reviewer #1: Yes

Reviewer #2: Yes

**********

3. Has the statistical analysis been performed appropriately and rigorously? 

Reviewer #1: N/A

Reviewer #2: Yes

**********

4. Have the authors made all data underlying the findings in their manuscript fully available?

The PLOS Data policy requires authors to make all data underlying the findings described in their manuscript fully available without restriction, with rare exception (please refer to the Data Availability Statement in the manuscript PDF file). The data should be provided as part of the manuscript or its supporting information, or deposited to a public repository. For example, in addition to summary statistics, the data points behind means, medians and variance measures should be available. If there are restrictions on publicly sharing data—e.g. participant privacy or use of data from a third party—those must be specified.

Reviewer #1: Yes

Reviewer #2: Yes

**********

5. Is the manuscript presented in an intelligible fashion and written in standard English?

PLOS ONE does not copyedit accepted manuscripts, so the language in submitted articles must be clear, correct, and unambiguous. Any typographical or grammatical errors should be corrected at revision, so please note any specific errors here.

Reviewer #1: Yes

Reviewer #2: Yes

**********

6. Review Comments to the Author

Please use the space provided to explain your answers to the questions above. You may also include additional comments for the author, including concerns about dual publication, research ethics, or publication ethics. (Please upload your review as an attachment if it exceeds 20,000 characters)

Reviewer #1: Thank you for the opportunity to review the revised manuscript. The revisions are thoughtful and thorough, and I appreciate the clear effort to address all reviewer comments.

The addition of structured subheadings in the Results section improves readability. Including a formal assessment of data saturation adds methodological rigor, and the updated reference list strengthens the manuscript’s relevance to current research.

This is a much-improved manuscript. All concerns have been addressed. Thank you again for your careful revision.

Reviewer #2: The suggested changes and coorections have been made to my satisfaction, thanks!

As a few similar studies have been done, it would be a fresh breeze to add what you suggest should be done to fill ths huge gap

**********

7. PLOS authors have the option to publish the peer review history of their article (what does this mean? ). If published, this will include your full peer review and any attached files.

If you choose “no”, your identity will remain anonymous but your review may still be made public.

**Do you want your identity to be public for this peer review?** For information about this choice, including consent withdrawal, please see our Privacy Policy .

Reviewer #1: No

Reviewer #2: **Yes: ** Dr Ina Diener

**********

# Editor report: Acceptance letter

**DOI:** 10.1371/journal.pone.0324859.r005

**Authors:** Tadashi Ito

PONE-D-24-37525R1

PLOS ONE

Dear Dr. Pike,

I'm pleased to inform you that your manuscript has been deemed suitable for publication in PLOS ONE. Congratulations! Your manuscript is now being handed over to our production team.

At this stage, our production department will prepare your paper for publication. This includes ensuring the following:

* All references, tables, and figures are properly cited

* All relevant supporting information is included in the manuscript submission,

* There are no issues that prevent the paper from being properly typeset

You will receive further instructions from the production team, including instructions on how to review your proof when it is ready. Please keep in mind that we are working through a large volume of accepted articles, so please give us a few days to review your paper and let you know the next and final steps.

Lastly, if your institution or institutions have a press office, please let them know about your upcoming paper now to help maximize its impact. If they'll be preparing press materials, please inform our press team within the next 48 hours. Your manuscript will remain under strict press embargo until 2 pm Eastern Time on the date of publication. For more information, please contact onepress@plos.org.

If we can help with anything else, please email us at customercare@plos.org.

Thank you for submitting your work to PLOS ONE and supporting open access.

Kind regards,

PLOS ONE Editorial Office Staff

on behalf of

Dr. Tadashi Ito

Academic Editor

PLOS ONE
